# Targeting *Enterococcus faecalis* HMG-CoA reductase with a non-statin inhibitor

**DOI:** 10.1038/s42003-023-04639-y

**Published:** 2023-04-03

**Authors:** Sucharita Bose, C. Nicklaus Steussy, Daneli López-Pérez, Tim Schmidt, Samadhi C. Kulathunga, Mohamed N. Seleem, Mark Lipton, Andrew D. Mesecar, Victor W. Rodwell, Cynthia V. Stauffacher

**Affiliations:** 1 Department of Biological Sciences, Purdue University, 915 West State Street, West Lafayette, IN 47907 USA; 2 Department of Chemistry, Purdue University, 560 Oval Drive, West Lafayette, IN 47907 USA; 3 Department of Comparative Pathobiology, College of Veterinary Medicine, Purdue University, 625 Harrison Street, West Lafayette, IN 47907 USA; 4 Department of Biochemistry, Purdue University, 175 South University Street, West Lafayette, IN 47907 USA; 5 Present Address: Institute for Stem Cell Science and Regenerative Medicine, GKVK Post Bellary Road, Bangalore, 560065 India; 6 Present Address: Food and Drug Administration, 10903 New Hampshire Avenue, Silver Spring, MD 20993 USA; 7 Present Address: Department of Biomedical Sciences and Pathobiology, VA-MD College of Veterinary Medicine, Virginia Tech, 205 Duck Pond Drive, Blacksburg, VA 24061 USA

**Keywords:** Enzyme mechanisms, Biophysical chemistry, Drug discovery

## Abstract

HMG-CoA reductase (HMGR), a rate-limiting enzyme of the mevalonate pathway in Gram-positive pathogenic bacteria, is an attractive target for development of novel antibiotics. In this study, we report the crystal structures of HMGR from *Enterococcus faecalis* (efHMGR) in the apo and liganded forms, highlighting several unique features of this enzyme. Statins, which inhibit the human enzyme with nanomolar affinity, perform poorly against the bacterial HMGR homologs. We also report a potent competitive inhibitor (Chembridge2 ID 7828315 or compound 315) of the efHMGR enzyme identified by a high-throughput, in-vitro screening. The X-ray crystal structure of efHMGR in complex with 315 was determined to 1.27 Å resolution revealing that the inhibitor occupies the mevalonate-binding site and interacts with several key active site residues conserved among bacterial homologs. Importantly, 315 does not inhibit the human HMGR. Our identification of a selective, non-statin inhibitor of bacterial HMG-CoA reductases will be instrumental in lead optimization and development of novel antibacterial drug candidates.

## Abstract (web summary)

High-throughput in vitro screening and crystal structures identify a non-statin inhibitor of HMG-CoA reductase for novel antibacterial drug design.

## Introduction

Antibiotic resistance in pathogenic bacteria is a pressing threat to human health worldwide. In recent years several Gram-positive bacteria including *Staphylococcus aureus* have acquired resistance to multiple antibiotics by lateral gene transfer events from *Enterococcus*^1,2^ rendering their clinical management difficult. The prevalence of methicillin resistant *Staphylococcus aureus* (MRSA) and vancomycin-resistant *Enterococcus* (VRE) is a major public health concern as infections caused by these antibiotic-resistant bacteria are associated with high morbidity and mortality rate^3^. Antibiotics currently in the market or the approval pipeline are either variants of the old classes or target the same molecular pathways and so fall prey to these same resistance mechanisms. The identification of alternative molecular targets is a necessity to overcome this resistance.

The mevalonate pathway is an attractive target for design of such novel antibacterials. It consists of a series of enzymes that takes three equivalents of acetyl-CoA and a sequence of various cofactors to produce isopentenyl diphosphate via a mevalonate intermediate. Isopentenyl diphosphate (IPP) is the central metabolic component of isoprenoids that are involved in numerous metabolic functions across the biological spectrum^4,5^. In bacteria, IPP produces undecaprenol, which is involved in the biosynthesis of the peptidoglycan cell wall^6^, and ubiquinones and menaquinones for the electron transport chain as well as carotenoids including staphyloxanthin in *S. aureus*^7^.

The necessity of the mevalonate pathway in the survival of low G + C Gram-positive bacteria, including *S. aureus*^8^*, Enterococcus faecalis* and *Streptococcus pneumoniae*^9^ has prompted several investigations into the pathway as a possible target for the development of a novel antibiotic. It should be noted that all Gram-negative and other Gram-positive bacteria do not produce IPP via the mevalonate pathway. Several bacterial species including *Escherichia coli, Haemophilus influenzae, Helicobacter pylori and Bacillus subtilis* utilize the non-mevalonate 1-deoxy-D-xylulose 5-phosphate/2-*C*-methyl-D- erythritol 4-phosphate (DOXP/MEP) pathway^10,11^. Thus, an inhibitor of the mevalonate pathway would specifically target the low G + C Gram-positive cocci, including major pathogenic bacteria that depend on this pathway, without affecting the normal host-gut flora, an approach that is being recognized as important for human health and healing^12^.

HMG-CoA reductase (HMGR) catalyzes the four-electron reduction of (*S)-*HMG-CoA to *(R)-*mevalonate, which is the rate-limiting step of the mevalonate pathway^13^. HMGR has also been shown to be one of the mevalonate pathway enzymes essential for survival in pathogenic bacteria, and hence is an attractive target for inhibitor design^14^. Sequence alignment and 3D structures reveal two evolutionary divergent classes of HMGR with eukaryotic and most archeal homologs grouped as Class I enzymes and the bacterial and few archeal homologs as Class II enzymes. Moreover, the substantial differences in the active site architecture between the human and the bacterial homologs, which is reflected by the 10^4^-fold difference in inhibition by the cholesterol lowering drug statins, enables the design of an inhibitor specific for the bacterial enzymes^14,15^.

In this pursuit, we report the crystal structure of the target, HMG-CoA reductase in the apo form (Ref-apo), and in complex with the ligands HMG-CoA, mevalonate and the cofactor NADP^+^ (Ref-ternary), from the pathogenic bacteria *E. faecalis*. In addition, an extensive in vitro screen was undertaken in search for small molecule inhibitors to this enzyme that identified a lead compound with an IC_50_ in the low micromolar range. Finally, the crystal structure (Ref-315) of this inhibitor (Chembridge2 ID 7828315) bound to the *E. faecalis* HMG-CoA reductase enzyme (efHMGR) was solved, detailing the molecular mechanism of inhibition and setting the foundation for future structure-based drug design research.

## Results and discussions

### Overall structure

The first three steps of the mevalonate pathway involve enzymes Acetoacetyl-CoA thiolase, HMG-CoA synthase and HMG-CoA reductase. Acetoacetyl-CoA thiolase catalyzes the condensation of two acetyl-CoA molecules to produce acetoacetyl-CoA. HMG-CoA synthase catalyzes the condensation of acetyl-CoA with acetoacetyl-CoA to form HMG-CoA. HMG-CoA reductase then catalyzes the conversion of HMG-CoA to mevalonate^14^. The forward reaction is driven by NADPH and HMG-CoA whereas the reverse reaction by CoA-SH, mevalonate and NADP^+^. The *mvaE* gene product in *E. faecalis* encodes for a fusion protein that harbors two distinct catalytic functions^16^. The N-terminal domain (1-378) harbors the Acetoacetyl-CoA thiolase activity while the C-terminal domain contains the HMG-CoA reductase activity. For our study this C-terminal domain (residues 381-803) was cloned from the full-length gene in the laboratory of Prof. Victor Rodwell at Purdue^17^.

**Fig. 1:**
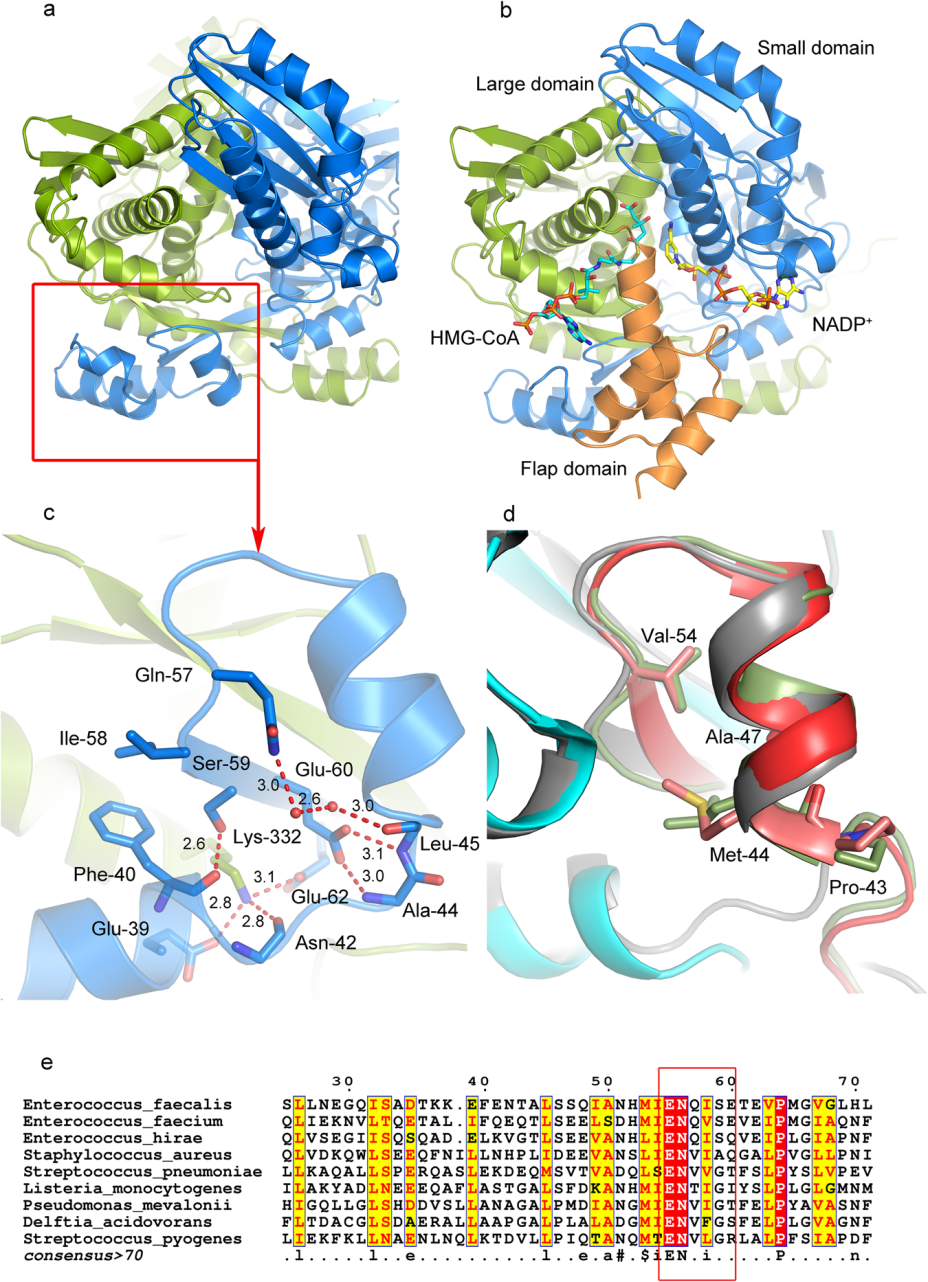
efHMGR dimer architecture and domain swap. **a** The apo efHMGR dimer structure: Two monomers interact in an intricate fashion to form the biologically active dimer, colored green and blue respectively. The N-terminal domain that is swapped is indicated by a red square. **b** In the liganded efHMGR dimer, the flap domain (orange) closes upon binding of HMG-CoA and NADP^+^ (represented as blue and yellow sticks). HMG-CoA primarily interacts with the large domain of one monomer (green) and NADP^+^ interacts with small domain (blue) of the other monomer. **c** The ENQISX_3_VP loop, unique in efHMGR, participates in various interactions at the crossover point. The interacting residues (Gln-39, Phe-40, Asn-42, Ala-44, Leu-45, Gln-57, Ile-58, Ser-59, Glu-60, Glu-62 and Lys-332) are represented in sticks. The H-bonds are shown as red dashed lines and the distances are shown in Å. **d** The ENVIG loop in pmHMGR (green) and DaHMGR (red) shown in cartoon representation suggest that presence of a proline residue preceding this loop along with other hydrophobic residues such as Met-44, Ala-47 and Val-54 prevent a complete domain swap. The dimer partner is shown in cyan. The “ENQIS loop” of efHMGR (grey) is shown as a reference. **e** Multiple sequence alignment of efHMGR with class II HMGR sequences from *S. pneumoniae*, *S. aureus*, *P. mevalonii*, *Listeria monocytogenes*, *Delftia acidovorans*, *E. faecium*, *E. hirae* show that the ENQIS loop (in red box) is unique to Enterococcus.

HMG-CoA reductase from *E. faecalis* (efHMGR) in apo and liganded forms was expressed, purified and crystallized (Methods). The crystal structures of the apo and the ligand bound enzymes were solved to 2.25 Å and 2.27 Å respectively. The resulting structures follow the overall architecture of the previously published bacterial homologs^18–21^. Briefly, efHMGR is an obligate, intertwined dimer with the two active sites composed from components from each of the monomers (Fig. 1a). In order to distinguish between residues originating from different monomers/chains, the chain ID is written in parenthesis next to the residue. For example, Asn-184 (A) means that the residue Asn-184 originates from chain/monomer A. Each monomer consists of three domains: a large domain (residues 1-108 and 212-370) that binds HMG-CoA in an extended conformation, a small domain (residues 109-211), comprised of a non-classical Rossmann nucleotide-binding domain that interacts with NADP^+^/NADPH, and finally a C-terminal flap domain (residues 371-423) that comprises the last 53 residues of the monomer (Fig. 1b). In Class II bacterial homologs^14^, the flap domain has a persistent 3-helical bundle architecture that interacts dynamically with the body of the protein as the reaction proceeds^18,20^.

Although the asymmetric unit of both the crystal forms (Ref-apo and Ref-ternary) consists of a tetramer arranged as dimer of dimers (AB and CD), the dimer-dimer interface is not conserved. In the apoenzyme structure, the dimer–dimer contact is primarily mediated by a calcium ion donated by calcium acetate in the crystallization condition. In the ligand bound complex, a different set of crystal-induced contacts are observed at the dimer-dimer interface involving the flap domain. Since the dimer-dimer interface is quite small and not conserved across different forms, it is likely a consequence of crystal packing and has no biological relevance (Supplementary Fig. 1 and Supplementary Note 1).

### efHMGR exhibits an N-terminal domain swap

A domain swap is a mechanism of oligomer formation wherein the monomers exchange similar structural elements between them, leading to higher order assembly^22^. The extent of exchange can range from a single secondary structural element to an entire tertiary domain. The biological role of a domain swap may be to provide a mechanism to regulate protein function, as well as an evolutionary strategy to form stable protein complexes^22,23^. HMG-CoA reductase exists in dimeric or higher oligomeric forms^19^ across species with enzymes that demonstrate different extents of domain swap of the N-terminal subdomain (residues 1-65)^15,19,21^. In the published Class I structures of human^24^ and archeal^25^ homologs, the entire sub-domain folds up against, crosses over and primarily interacts with the C-terminal domain of the adjacent monomer. However, in all of the Class II prokaryotic reductases solved so far, only 16 residues around the dimerization element ENVXGX_3_I/L/VP (residues 46-61) are swapped, while the rest of the N-terminal subdomain doubles backs on itself and interacts with the C-terminal domain of the same monomer^21,25,26^. HMGR from *E. faecalis* is found to be an exception. Though otherwise a classic Class II HMGR, its entire N-terminal domain (residues 1-65) undergoes a complete domain swap around the hinge region (residues 42-45) and interacts with the main domain of its dimer partner (Fig. 1a, c) and in doing so resembles the Class I homologs (Supplementary Fig. 2).

Several factors affect the extent of the domain swap in proteins, which includes the presence of proline residues as well as flexibility and the length of the hinge region around which the domain swaps^27^. Our data suggest that the “dimerization element” between αC and βA in efHMGR, which forms a β-sheet with the adjacent monomer, has an important role in the domain swap. Uniquely, the aforementioned element contains a sequence EN**Q**_57_I**S**_59_X_3_VP with substitutions glutamine and serine (underlined) for valine and glycine, otherwise present in most other bacterial homologues (Fig. 1e). All the three efHMGR structures reported here suggest that Gln-57, Ser-59 (in the ENQIS loop) and Glu-60 (residue X) make several interactions, which stabilizes this crossover (Supplementary Note 2). Hydrogen bond (H-bond) interactions between Phe-40, Asn-42, Ala-44, Leu-45, Gln-57, Ser-59, Glu-60, Glu-62 from one monomer and Lys-332 from the other monomer (of the dimer pair), around the cross-over point, stabilize the domain swap (Fig. 1c).

In other prokaryotic HMGR enzymes from *Pseudomonas mevalonii* (pmHMGR, PDB: 4i64)^20^, *S. pneumoniae* (SpHMGR, PDB: 5wpj)^18^, *Delftia acidovorans* (DaHMGR, PDB: 6eeu)^26^ and *Burkholderia cenocepacia* (BcHMGR, PDB: 6p7k)^19^, the ENQIS sequence is substituted by the ENVXG sequence which makes no such interactions (Fig. 1d and Supplementary Note 2).

This difference in the domain swap in the efHMGR structure could have a functional significance in the interactions with the fused Acetoacetyl-CoA thiolase domain. In this context, the efHMGR is similar to the Class I human HMGR which also possesses a large N-terminal membrane spanning domain responsible for timely degradation of the enzyme^28^. For efHMGR, stability of the dimer may be the functional role of the swap. Both *E. faecium* and *E. hirae* homologs, which encode the fusion protein with acetoacetyl-CoA thiolase and reductase activity in a single polypeptide^14,16^, display the “ENQI/VS” sequence as well (Fig. 1e).

### Active site architecture and conformational changes associated with ligand binding

**Fig. 2:**
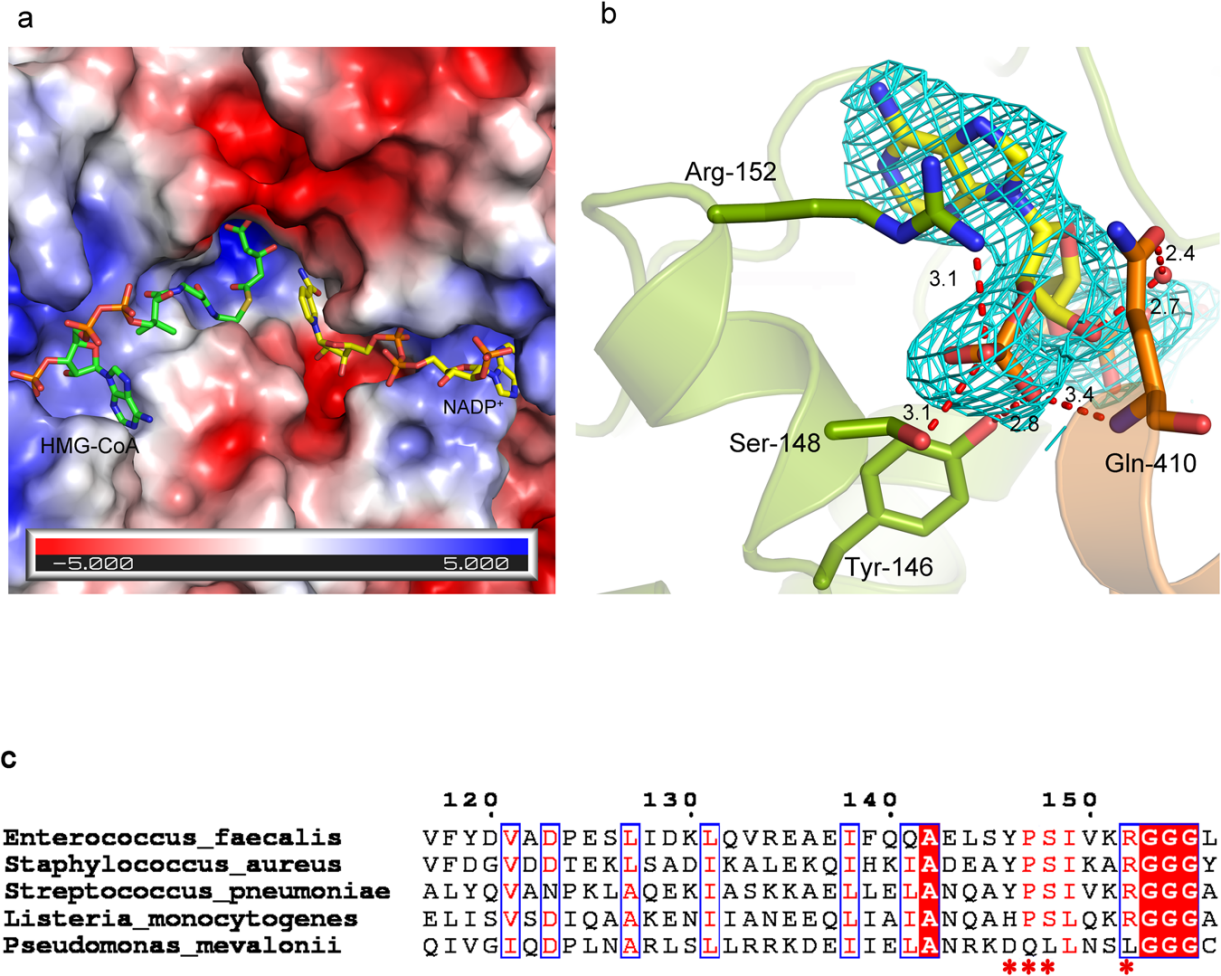
Structural determinants of NADP^+^ in efHMGR. **a** Electrostatic surface potential map of efHMGR with NADP^+^ and HMG-CoA bound in the active site. Both HMG-CoA (green) and NADP^+^ (yellow) are represented as sticks. **b** Tyr-146, Ser-148 and Arg-152 residues represent the molecular determinants present in the small domain of efHMGR that interact with the oxygen atoms of the 2'-ribose-phosphate group of NADP^+^. Gln-410 originating from the flap also interacts with NADP^+^. The NADP^+^ molecule is encased in a 2Fo-Fc difference density at 1.0σ. Hydrogen bond interactions are represented as red dashes and the distances are shown in Å. **c** Class II HMGRs of *S. pneumoniae, S. pyogenes, S. aureus and E. faecalis* utilize NADPH as the cofactor. The residues (marked with an asterix) that determine the cofactor specificity are conserved in these bacterial enzymes including efHMGR. The *P. mevalonii* HMGR, which utilizes NADH has Asp-146, Leu-148 and Leu-152 as the molecular determinants.

The active site present at the dimer interface of efHMGR is an open groove that forms a V shape along the surface of the molecule. One arm of the V harbors HMG-CoA while the other NADP^+^. The ternary complex structure shows that HMG-CoA binds to the large domain of one monomer and NADP^+^ to small domain of the other monomer (Figs. 1b and 2a). At the point of the V, the two monomers bring the ligands together in a deeper pocket that binds the six carbon HMG or mevalonate. This arrangement positions the HMG-CoA thioester carbonyl carbon in proximity to the C4 atom of the nicotinamide ring of NADPH for hydride transfer. A comparison of the efHMGR active site with other published prokaryotic HMGR enzymes (r.m.s. deviation ~0.1–0.3 Å) suggests this is a highly conserved active site with similar interactions observed between the ligands and the enzyme residues. The catalytic triad of Glu-86, Lys-263 and Asp-279 is arranged around the carbonyl oxygen of the thioester, ready to stabilize the developing negative charge of the intermediate and provide the hydrogen for the final conversion of mevaldehyde to mevalonate^29^. The interactions between the enzyme and the ligands, HMG-CoA, mevalonate and NADP^+^ are detailed in the supplementary section (Supplementary Fig. 3a–c).

The flap domain (residues 371-421) closes upon ligand binding, covering the active site and bringing the catalytic His-376 into position. The hinge region preceding the flap domain in efHMGR (residues 368-370) undergoes substantial conformational change upon ligand binding. In the apoenzyme, Glu-370, the last residue of the hinge is partially disordered. However, upon ligand binding, the backbone of Glu-370 reorients (ΔΦ = −78.1° ΔΨ = −12.9 °) such that the side chain carboxyl group of Glu-370 makes salt bridge interactions with the guanidinium group of Arg-365. Glu-370 further connects to the diphosphate moiety of HMG-CoA via water mediated hydrogen bond interaction. The guanidino group of Arg-365 interacts with the carbonyl oxygen of the pantothenic moiety of HMG-CoA through a water molecule. Additionally, Glu-370 interacts with the subsequent residues, Gly-371 and Gly-375 present in the first helix of the flap via a water molecule (Supplementary Fig. 4). The salt bridge interaction between Arg-365 and Glu-370 is unique to efHMGR and is observed every time the flap is ordered suggesting that it could be important for the flap domain closure. These residues, although present in pmHMGR, do not engage in such interactions in the closed flap structures (PDB: 1qax, 4i4b).

In the ternary complex structure of efHMGR, each of the dimers (AB and CD) in the asymmetric unit harbors two active sites for a total of four. NADP^+^ is present in all the active sites and HMG-CoA in only one where the combination of HMG-CoA and NADP^+^ forms a non-active combination of ligands. Density consistent with mevalonate is also found in one of the active sites. This is not an uncommon observation, as HMG-CoA is known to undergo spontaneous reduction to mevalonate due to radiation damage (PDB: 1r31)^20^.

### Molecular determinants that specify NADP^+^ binding in efHMGR

NADH serves as the coenzyme for oxidative processes that yield ATP, whereas NADPH serves as the coenzyme for reductive biosynthetic reactions. As compared to NADH, NADPH harbors a phosphate group on the 2' -hydroxyl group of the ADP-ribose moiety, which separates these two pools of redox agents. Analysis of multiple dehydrogenases containing the classic Rossmann dinucleotide-binding domain suggests that a loop containing a GXGXXG/A fingerprint facilitates the binding of the enzyme to the adenine-ribose moiety of NADH/NADPH^30^. However, the selection of NADPH vs. NADH appears to depend on the residues surrounding this consensus sequence and varies between different families of dehydrogenases.

The specificity of the HMGR enzyme towards NADH or NADPH differs between the two classes. While the Class I eukaryotic homologs exclusively utilize NADPH, the Class II prokaryotic homologs can utilize either NADH or NADPH. The Class II reductase from *Archaeoglobus fulgidus* can exploit both the cofactors^31^, but pmHMGR is highly selective towards NADH^32^ and enzymes from *S. pneumoniae, S. aureus* and *E. faecalis* are specific towards NADPH^16^. In fact, efHMGR showed no activity when NADH was used and a ten-fold excess of NADH over NADPH could not inhibit the reaction^16^.

The homologs like pmHMGR, which are specific towards NADH, have a negatively charged amino acid, an invariant aspartate residue (Asp-146) on a short alpha helix (αF helix) in the small domain that interacts with the 2'- and 3'-hydroxyl group of the adenine-ribose moiety. Friesen et al.^33^ successfully improved the relative specificity of the pmHMGR enzyme towards NADPH by mutating Asp-146 to Ala and introducing a positively charged residue (Arg) in place of the neighboring Leu-148. These observations led them to hypothesize that a small residue at this position would provide extra room for the phosphate moiety and a nearby positively charged residue would neutralize the negative charge on the phosphate^33^.

Structural superposition of the pmHMGR enzyme (PDB: 1qax) over efHMGR, bound to their respective NAD/P cofactors shows that rather than selecting a smaller side chain at position 146 to make room for the phosphate, a large and bulky tyrosine residue (Tyr-146) was selected in efHMGR. This forces the adenine-ribose-phosphate up at close to a 90° angle compared to the *P. mevalonii* structure. The side chain hydroxyl group of Tyr-146 (D) then hydrogen bonds to the adenine-ribose-phosphate oxygen (O2X). The next residue in efHMGR is Pro-147 (D), which introduces a bend in the alpha-helix, thereby bringing the next residue, Ser-148 (D) into hydrogen bonding distance of the adenine-ribose-phosphate oxygen (O3X) (Fig. 2b). The two equivalent residues in pmHMGR are Gln-147 and Leu-148, neither of which can interact with the ribose hydroxyls. The final change is the Leu-152 residue in *P. mevalonii*, which makes VDW contact with the adenine base (3.6 Å) and is substituted by an Arginine residue (Arg-152) in *E. faecalis*. Arg-152 (D) forms a stacking interaction with the adenine base (3.5 Å) and also hydrogen bonds with the ribose-phosphate oxygen (O3X) of NADP^+^ (Fig. 2b). These four changes (D146Y, Q147P, L148S, and L152R) are conserved in Staphylococcal, Enterococcal and Streptococcal HMGR aligned sequences (Fig. 2c).

The previously published structure of SpHMGR (PDB: 5wpj)^18^ reported the interactions between the ribose-phosphoryl oxygens of NADPH and the analogous Ser and Arg residues, and suggested that Tyr-144 (analogous to Tyr-146 in efHMGR) forces the adenosine moiety away so that the ribose-phosphoryl oxygens can H-bond with Ser-146 (analogous to Ser-148 in efHMGR)^18^. Our efHMGR crystal structure shows that Tyr-146 accomplishes a dual role and not only forces the ribose-phosphate up so that it interacts with Ser-148 and Arg-152, but at the same time hydrogen bonds with the ribose-phosphoryl oxygen of NADP^+^ (O2X). Additionally, in efHMGR, the main chain amide group of Gln-410 (C), present in the loop between the 2nd and the 3rd helix of flap domain is at a potential hydrogen bond distance (3.4 Å) from the ribose-phosphate oxygen of NADP^+^ (O3X). A water mediated interaction is observed between the adenine-3'-ribose-hydroxyl moiety and the side chain carboxamide group of Gln-410. (Fig. 2b). These interactions were not observed in SpHMGR as the flaps in these structures were positioned in an open configuration distant from the active site^18^.

### Role of NADP^+^ in flap domain closure

In HMGR enzymes, the C-terminal flap domain that closes over the active site upon ligand binding plays a crucial role in catalysis. In the Class I (archeal and eukaryotic) homologs^34^, it is relatively short and consists of only the first helix of the larger three-helical bundle characteristic of the Class II bacterial enzymes^15^. Although the significance of this larger flap domain is not clear, the published crystal structures demonstrate that the bacterial three-helical bundle undergoes several conformational rearrangements during ligand entry, exit and catalysis^18,20,32^ (Supplementary Note 3).

There is agreement on a general scheme for the steps in the complex reaction mechanism catalyzed by both the human and the bacterial HMG-CoA reductases. Initially the binding of both HMG-CoA and NADH/NADPH induces flap domain ordering and closure over the active site. Next, the thioester of HMG-CoA is reduced by the first hydride transfer and the resulting oxyanion is stabilized by catalytic Glu-83 and Lys-267 (pmHMGR nomenclature). Early studies demonstrated that the resulting thiohemiacetal is the predominant intermediate although it is in dynamic equilibrium with the aldehyde and thioanion^35,36^. The flap domain changes configuration that allows the release of the oxidized NAD^+^/NADP^+^. This leads to binding of another molecule of NADH/NADPH followed by rapid reduction of the aldehyde to the product, mevalonate assisted by catalytic Glu-83. The catalytic His-381 donates a proton to the thioanion forming the second product CoA-SH. The flap again changes configuration allowing the exit of these products and the second oxidized NAD^+^/NADP^+^. Details of some of these steps have been confirmed in recent molecular dynamics studies^29^.

**Fig. 3:**
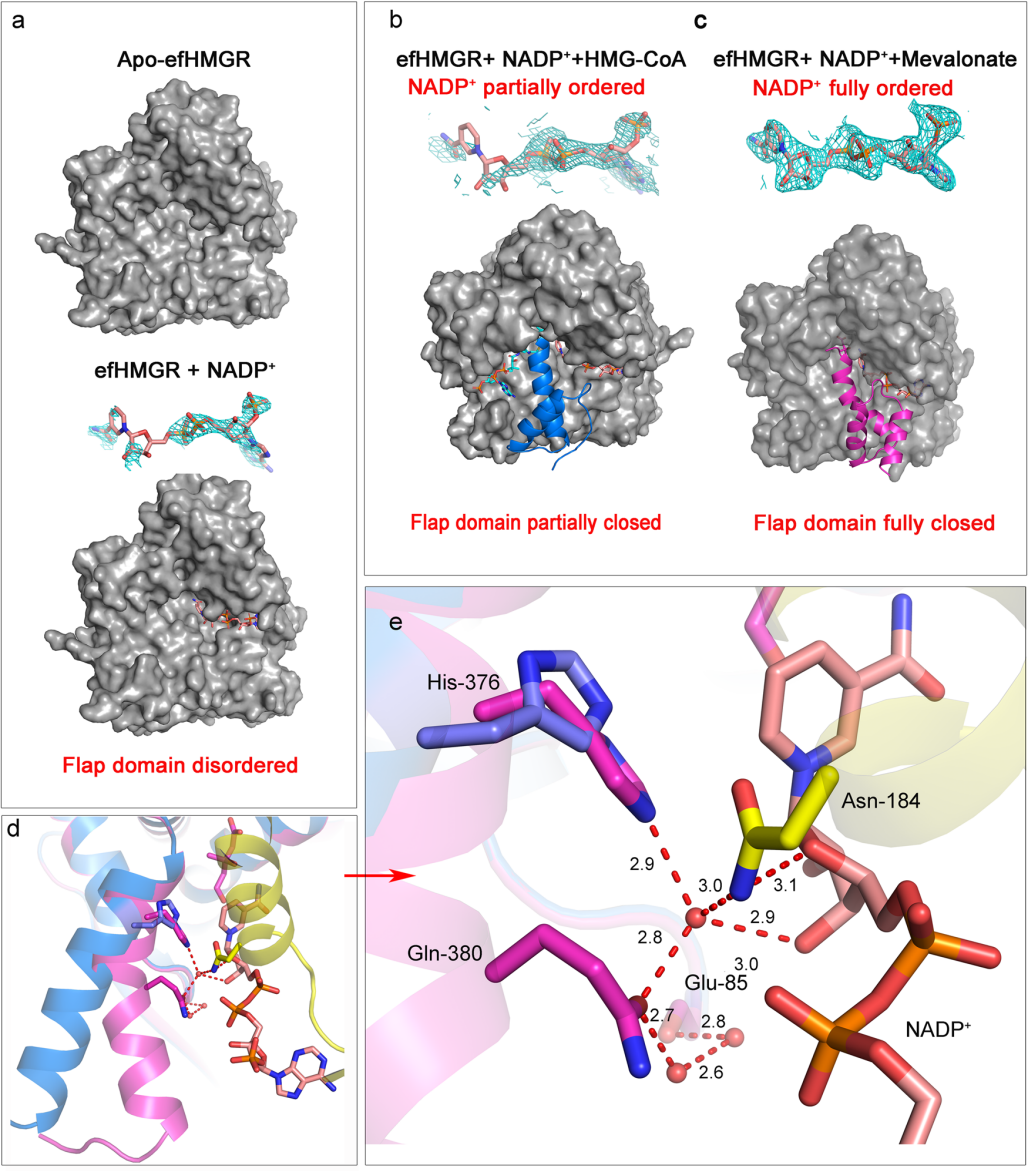
Role of NADP+ in flap domain closure. **a** The flap domain is disordered in the apo and NADP^+^ bound forms in efHMGR. With only NADP^+^ bound in the active site, the flap domain is disordered and the nicotinamide- ribose moiety in NADP^+^ does not have observable 2Fo-Fc density. **b** In the presence of HMG-CoA and NADP^+^, the flap domain is partially closed (blue). In this case the nicotinamide-ribose moiety of NADP^+^ is also disordered as seen by the 1.0σ 2F_o_-F_c_ density_._ **c** The flap domain is fully closed (magenta) in the presence of mevalonate and a fully ordered NADP^+^ molecule as evident by the  2Fo-Fc density at 1.0σ. **d** Superposition of the HMG-CoA + NADP^+^ bound monomer on the mevalonate + NADP^+^ bound monomer suggests that the partially closed flap domain (blue) is rotated 15° as compared to the fully closed conformation (magenta). **e** The interactions that stabilize the fully closed flap (magenta) in the presence of a well-ordered NADP^+^ molecule are shown here. Several residues (His-376 and Gln-380) in the fully closed flap interact with the nicotinamide-ribose moiety of NADP^+^ via water-mediated interactions. Residue Asn-184 present in the small domain is seen to clamp down over the nicotinamide-ribose moiety and also participate in the water-mediated interactions. The catalytic His-376 is placed farther away in the partially closed flap (blue). Waters are shown as red spheres and hydrogen bond interactions are represented as red dashes and the distances are shown in Å.

The liganded efHMGR structure (Ref-ternary) reported here suggests that the flap is disordered when NADP^+^ alone is present in the active site. In these structures, a combination of either HMG-CoA + NADP^+^ or mevalonate + NADP^+^ needs to be present for the flap to close. (Fig. 3a–c). However, a closer look indicates that the flap domain has a different conformation (r.m.s deviation of Cα ~ 1.4 Å) in these two ligand combinations (Fig. 3d). With mevalonate and NADP^+^ in the active site (Dimer CD), the flap domain is seen in the fully closed conformation with His-376 (C) at hydrogen bond distance from the catalytic Ser-88 (D). The 2Fo-Fc electron density map shows a well-ordered NADP^+^ molecule with B factors comparable to the surrounding residues. The carboxamide group of the nicotinamide ring hydrogen bonds with the side chain of Asn-213 (D). Also, the carboxamide group of Asn-184 (D) hydrogen bonds (3.1 Å) with the nicotinamide ribose-oxygen and clamps the cofactor down in the active site. Several direct and water mediated hydrogen bond interactions connect the cofactor with the flap domain of the enzyme. While the nicotinamide-ribose 2'-OH hydrogen bonds with Asn-279 (C) (2.9 Å) of the large domain, the nicotinamide-ribose 3'-OH also interacts with the catalytic His-376 (C) via a well-ordered and multiply H-bonded water molecule (His-376 (2.9 Å), Gln-380 (C) (2.8 Å), ribose-3'-OH (2.9 Å) and Asn-184 (D) (3.0 Å)). One turn further down this helix, Gln-380 (C) further connects with Glu-85 (C) via two water-mediated hydrogen bonds. This web of interactions serves to keep the catalytic His-376 near Ser-88 and the thioester moiety of HMG-CoA (Fig. 3e). As seen in other bacterial reductases, further down the first helix in the flap domain, Met-386 (C) engages in hydrophobic interactions with Met-53 (D) and Val-329 (D) and mediates interaction with the main body of the enzyme. In the published ternary complex structures of pmHMGR (PDB: 4i4b, 1qax)^20,32^, a well-ordered NAD^+^ also facilitates similar interactions with His-381 and keeps the flap domain in a fully closed conformation. Miller et al.^18^ observed that in the apo-SpHMGR and NADPH bound SpHMGR structure the flap is ordered but adopts an open conformation. In this conformation the flap is distant from the substrate and cofactor binding site^18^. Their observation is different from both efHMGR and pmHMGR wherein the flap is not observed unless both the ligand and the cofactor are bound. Thus, it is highly likely that in the absence of ligands, the flap domain is flexible and can attain multiple open conformations that may be unresolved in apo-HMGR structures.

However, with HMG-CoA and NADP^+^ in the active site (Dimer AB), the flap domain in efHMGR is observed in a “partially closed” conformation, similar to the HMG-CoA bound SpHMGR structure (PDB: 5wpk)^18^. The flap is rotated 15° compared to the canonical *pm*HMGR flaps (PDB: 4i4b, 1qax)^20,32^ and this rotation displaces and reorients the active site histidine (His-376) from its site of action near Ser-88 and the CoA sulfhydryl group. Additionally, the NADP^+^ molecule is only well ordered from the adenine-ribose until the pyrophosphate moiety while the nicotinamide and the nicotinamide-ribose moiety are disordered as evident by high B factors (~65–70 Å^2^) and lack of an observable 2Fo-Fc density. As a result, no interaction is seen between the flap domain and the nicotinamide-ribose moiety of NADP^+^, thereby making the flap relatively flexible to orient itself in a different conformation exposing the cofactor binding site (Fig. 3b, d). We do recognize that the partially closed conformation could also have resulted from crystal packing as may be the case in most of the other bacterial homologs. However, this conformation of the flap does provide the structural evidence for its flexibility during the course of the reaction.

The cofactor exchange step following the first hydride transfer wherein the oxidized NADP^+^ exits the active site followed by binding of a reduced equivalent is crucial for catalysis. Several structures from various bacterial homologs suggest that the flap domain alternates between multiple conformations to facilitate this cofactor exchange. Our structural data shows that both the substrate and the cofactor sites need to be occupied for the flap to close. However, the extent of the closure of the flap domain and the position of His-376 with respect to Ser-88 is dependent on the interaction between the nicotinamide-ribose of NADP^+^ and the first helix of the flap domain. An ordered nicotinamide–ribose 3'-OH group in NADP^+^ facilitates interactions with several residues of the flap including the catalytic His-376 via water-mediated hydrogen bond network. This depicts the fully closed conformation as seen previously in the pmHMGR abortive complex and productive complex. However, when the nicotinamide-ribose group is disordered, which represents the situation where the cofactor is ready to exit the active site, elimination of this H-bond network frees the flap domain to reorient itself in a partially closed conformation that leaves the cofactor-binding site open and accessible. Our data supports the hypothesis made by Miller et al.^18^ who also visualized the partial closure of the flap in the presence of HMG-CoA alone and suggested that after the first hydride transfer, the flap domain moves away from the NADP^+^ binding site, which permits the cofactor exchange while keeping the intermediates bound to the enzyme.

### Identification of a novel inhibitor against efHMGR

**Fig. 4:**
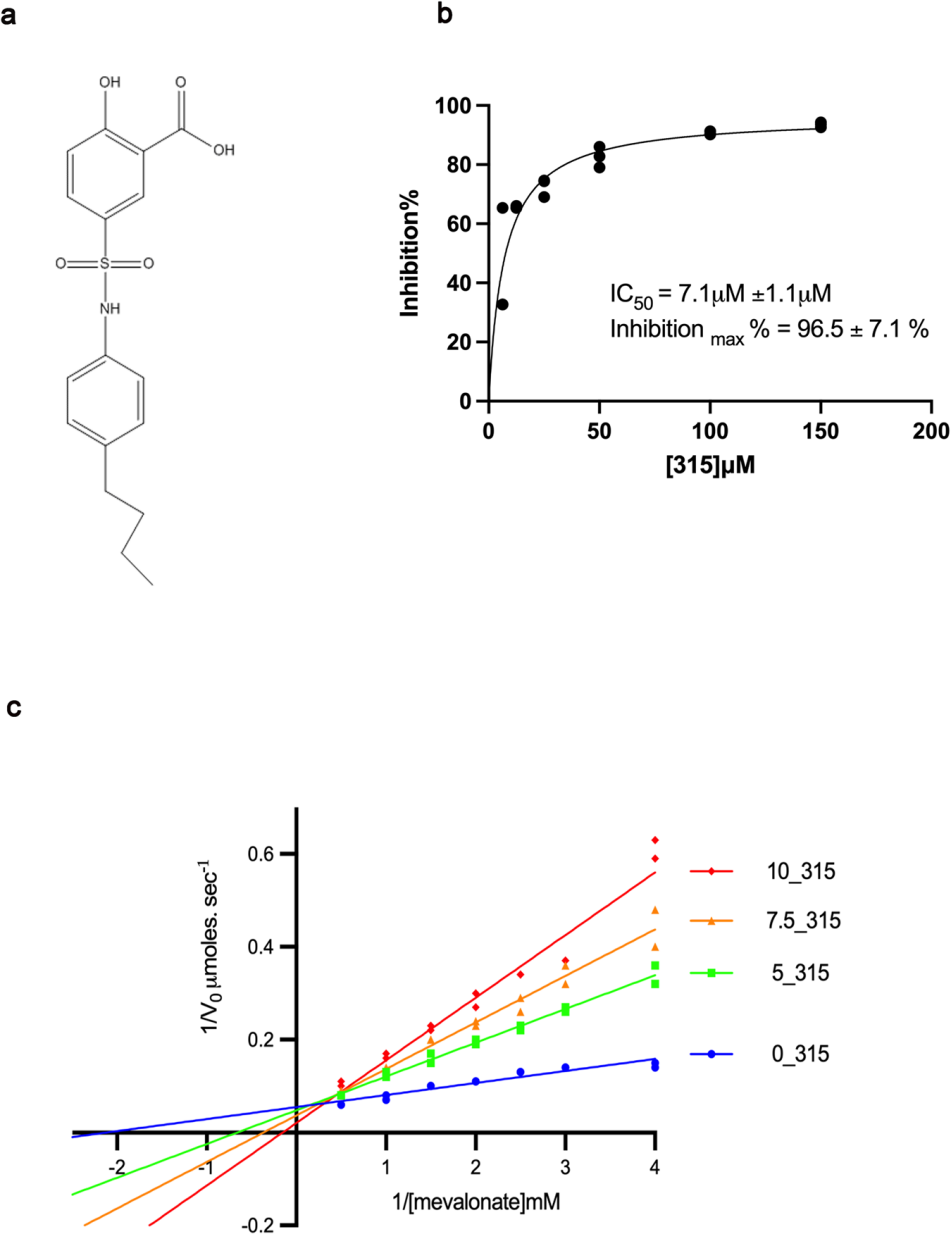
315 is a competitive inhibitor of efHMGR. **a** Chemical structure of 315 (Chembridge2 ID 7828315): 5-{[(4-butylphenyl) amino] sulfonyl}-2-hydroxybenzoic acid. This compound is composed of a hydrophobic 4-butylphenyl group linked to the 2-hydroxy benzoic acid via an amino and a sulfonyl group. **b** Dose-response curves of 315 against *E. faecalis* HMG-CoA reductase. The IC_50_ value was estimated by Michaelis-Menten fitting of the inhibition % vs. inhibitor concentration data. Assays were conducted in triplicates **c** A Lineweaver plot shows that 315 is a competitive inhibitor of efHMGR. An analysis of this plot indicates that 315 inhibits the conversion of mevalonate to HMG-CoA with a Ki of 2 μM. Assays were conducted in duplicates and in the presence of 0, 5, 7.5 and 10 μM of 315 under otherwise standard conditions. Both datasets  were analyzed by Graphpad Prism 9.0 and presented as individual data points.

In collaboration with Southern Research Institute (SRI), a library of 300,000 compounds from the Chembridge 2, SRI and Molecular Libraries Screening Center Network (MLSCN) database were screened using a high-throughput assay (Methods). Inhibitory activity was ascertained in a dose-response screen to determine IC_50_ values. The assay involved the oxidation reaction of mevalonate to HMG-CoA where the conversion of NADP^+^ to NADPH was measured spectrophotometrically at 340 nm. Twenty six of the inhibitors were selected for further testing at Purdue. One of the compounds from the Chembridge 2 panel, ID 7828315, hereafter referred to as 315 (Fig. 4a) was found to potently inhibit efHMGR with an IC_50_ value of 7.1 μM (Fig. 4b). The kinetic mechanism of inhibition for compound 315 was next determined in the mevalonate to HMG-CoA direction by varying the concentration of mevalonate at fixed, variable concentrations of 315. The results shown in Fig. 4c indicate that compound 315 is a competitive inhibitor against mevalonate for efHMGR with a resulting K_i_ value of 2.0 μM. Compound 315 appears to be specific towards Class II HMGR as it does not show any inhibition of the Class I human enzyme in this reaction at a concentration of 100 µM.

### Molecular mechanism of inhibition of efHMGR by 315

**Fig. 5:**
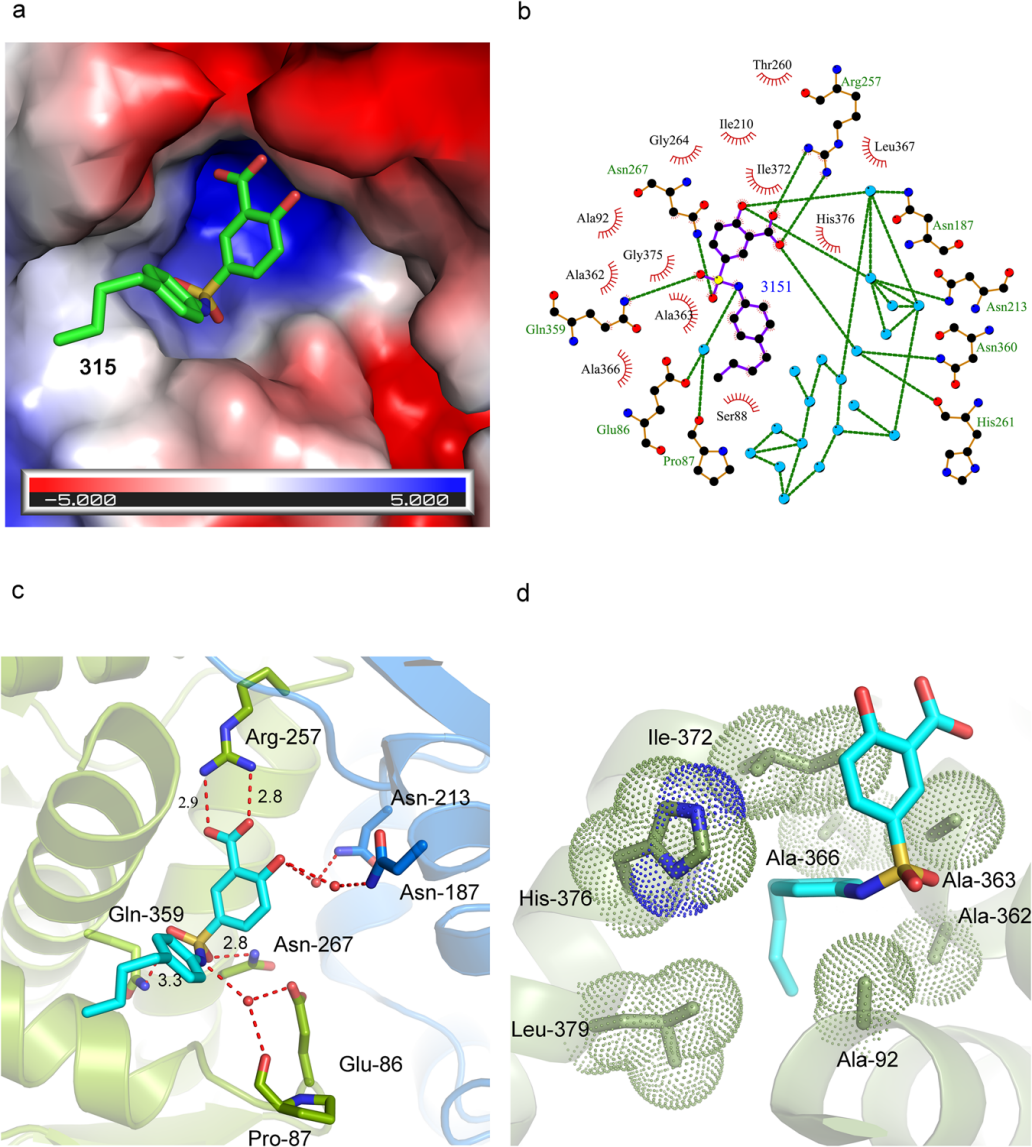
315 binds in the mevalonate binding pocket in efHMGR. **a** Electrostatic potential map showing 315 binding in the mevalonate binding pocket present at the dimer interface. The benzoic acid moiety is tucked inside a pocket lined with charged residues while the butylphenyl group of 315 is accommodated in a hydrophobic environment. **b** Ligplot diagram showing interactions between 315 and the mevalonate pocket residues in efHMGR. Hydrogen bond interactions are represented by green dashed lines, waters as cyan spheres and hydrophobic contacts as brown semicircles. **c** Hydrophilic interactions stabilize 315 in the mevalonate binding pocket of efHMGR. The large domain residues, Glu-86, Pro-87, Arg-257, Asn-267 and Gln-359, colored green, interact with the ligand via direct and water-mediated hydrogen bond interactions. The small domain residues from the adjacent monomer, Asn-187 and Asn-213, colored blue, also interact with the ligand via water molecules. Red spheres represent water molecules. Red dashes represent the hydrogen bond interactions with the numbers indicating the hydrogen bond distances in Å. **d** Hydrophobic interactions also stabilize 315 in the active site. Hydrophobic residues such as Ala-92, Ala-362, Ala-363, Ala-366, Ile-372 and His-376 pack against the hydrophobic butylphenyl group and stabilize the ligand. The residues are represented as sticks with Van der Waals radii as dotted spheres.

Compound 315, 5-{[(4-butylphenyl)amino] sulfonyl}-2-hydroxybenzoic acid (Fig. 4a), is composed of a hydrophobic 4-butylphenyl group linked to the 2-hydroxy benzoic acid via a sulfonamide group and mimics a number of interactions of the natural mevalonate/HMG-CoA substrate. In order to examine the molecular details of the inhibition of efHMGR by 315, a complex of the inhibitor with efHMGR was crystallized (Methods). These crystals proved to be far better ordered than the apo or liganded forms and produced a high resolution structure that was solved to 1.27 Å (Table 1, Ref-315). The unambiguous electron density of 315 in the active site of efHMGR demonstrated the structural features of 315 that are important for binding. The 2-hydroxy benzoic acid moiety of 315 primarily occupies the deep-seated mevalonate-binding pocket of the enzyme (Fig. 5a), replicating some of the binding interactions of the mevalonate substrate. The amino-sulfonyl butylphenyl group then extends down the largely hydrophobic cleft otherwise occupied by the CoA moiety of HMG-CoA. A superposition of the HMG-CoA/mevalonate containing structure with the 315 inhibited structure shows this compound occupies a similar space as the natural substrate. Thus, the structural data supports the kinetic data that 315 is a competitive inhibitor of mevalonate oxidation with K_i_ value of 2 μM (Fig. 4c).

**Table 1:** Data collection and refinement statistics.

	Ref-apo	Ref-ternary	Ref-315
PDB	7M66	7M1Z	7M3H
Ligands added	–	HMG-CoA and NADP^+^	Compound 315
Data collection			
Space group	P6_1_	C2	P2_1_2_1_2_1_
Cell dimensions			
*a*, *b*, *c* (Å)	168.7, 168.7, 120.2	241.3, 62.6, 171.9	72.5,81.1,153.0
α, β, γ (°)	90, 90, 120	90, 133.5, 90	90,90,90
Resolution (Å)	50-2.25 (2.33-2.25)	39.5-2.27 (2.35-2.27)	44-1.27 (1.31-1.27)
*R*_merge_	11.6 (66.5)	8.8(44.4)	6.3(96.6)
*I*/σ*I*	16.5(2.5)	15.8(2.1)	23.7(1.9)
Completeness (%)	99.9(99.8)	88.9(61.7)	99.9(99.7)
Redundancy	6.5 (1.9)	5.8 (4.1)	5.2(5.1)
Refinement			
Resolution (Å)	2.25	2.27	1.27
No. reflections	91404	76031	237117
*R*_work_/*R*_free_	17.5/20.9	19.6/23.9	15.2/16.6
No. atoms			
Protein	11153	12137	650
Ligand/ion	287	578	105
Water	646	340	973
*B*-factors			
Protein	46.6	58.5	17.3
Ligand/ion	57.7	61.1	18.3
Water	43.5	48.8	31.6
R.m.s. deviations			
Bond lengths (Å)	0.002	0.008	0.008
Bond angles (°)	0.557	0.873	0.978

Values in parentheses refer to the highest resolution bin.

Several direct and water-mediated hydrogen bond interactions stabilize the inhibitor in the active site (Table 2). Two direct hydrogen-bond interactions are observed between the carboxylate group of the benzoic acid moiety of 315 and the guanidinium group of Arg-257(A), located deep inside the active site pocket (Fig. 5b, c). This interaction is analogous to that between the carboxylic group of HMG/mevalonate and Arg-257. The sulfonyl oxygen of the linking sulfonyl amino group makes a hydrogen bond interaction with the Asn-267 (A) side chain amide group. A potential weak hydrogen bond interaction is also observed between the other sulfonyl oxygen and the side chain amide group of Gln-359 (A).

**Table 2:** List of hydrogen bond interactions between 315, the efHMGR enzyme and water molecules.

315 atoms	Protein/water	Distance (Å)
O01	Arg-257(A)	2.8
O24	Arg-257 (A)	2.9
O01	Water-790	2.9
O05	Water-861	2.9
O22	Asn-267(A)	2.8
O10	Gln-359 (A)	3.3
N11	Water-750	3.0

Several hydrophobic interactions also stabilize the inhibitor in the active site. The benzoic acid moiety of 315 is stabilized by hydrophobic interactions with residues Leu-367 (A) and Ile-210 (B). The butylphenyl moiety of 315 is accommodated in the hydrophobic cleft near the surface of the enzyme and stabilized by close hydrophobic contacts (4.0–5.0 Å) with residues Ala 362, Ala-363 and Ala-366 present in the long α-helix (residues 350-370) that precedes the flap domain. Ile-372 (A) present in the loop preceding the flap domain nestles into the bend of the inhibitor (Fig. 5b, d).

Additionally, several structural water molecules line the active site and form a hydrogen-bond network that connects the inhibitor and the enzyme. A water molecule mediates a hydrogen bond interaction between Asn-187 (B) of the small domain of efHMGR and the 2-hydroxy group of the benzoic acid moiety of 315. A second water connects the carboxylate of the benzoic acid moiety of 315 with the side chain amide group of Asn-360 (A) and main chain carbonyl oxygen atom of His-261(A) from the large domain of the enzyme (Fig. 5b). A similar interaction is also noticed between the carboxyl group of HMG-CoA and Asn-360 in other Class II structures. The amino group of 315 interacts with the active site residues, Glu-86 and Pro-87 via a third water molecule (Fig. 5b, c). These water-mediated interactions indicate the potential sites in the inhibitor that could be modified in the lead optimization step to further increase its affinity towards efHMGR (Fig. 5b, c).

The three helices of the flap are ordered and the first helix is seen to partially close over the active site in the presence of 315. The absence of NADP^+^ in the active site of the 315 bound structure precludes necessary interactions, which are important for the flap to fully close. Similar conformation of the flap was observed in the ternary efHMGR structure in the presence of HMG-CoA and a partially ordered NADP^+^. The butylphenyl group of 315 is well accommodated in the hydrophobic pocket formed by the residues from the large domain as well as the flap domain from the same monomer. Ile-372, His-376 and Leu-379 present in the first helix of the flap line the hydrophobic pocket on one side whereas several residues from the large domain (of the same monomer) including Ala-92, Ala-362 and Ala-363 constitute the other side (Fig. 5d). The partial closure of the flap is achieved by the hydrophobic interactions between the butylphenyl group of 315 and the flap, and may interfere with NADPH binding during catalysis.

An earlier study reported several inhibitors against SpHMGR which were identified through virtual screening and also validated by molecular docking, site-directed mutagenesis and enzymatic assays. These compounds inhibited the SpHMGR with a low micromolar IC_50_ and targeted the joint space between HMG-CoA and NADPH. The 3-amino 1,2,5 oxadiazole moiety of the compound 4 occupied the NADPH binding pocket and interacted with the invariant Asn-212 and the hydrophobic phenyl group occupied the HMG-CoA binding site^37^. Thus, a strategy for lead optimization would be to extend 315 beyond the HMG-CoA binding pocket such that it also occupies the NADPH binding pocket, thereby increasing the inhibitor potency and specificity.

### 315 poorly inhibits the Class I eukaryotic homolog but shows potency against efHMGR as compared to the statins

While 315 inhibits efHMGR with a single digit micromolar (7.1) IC_50_, it completely fails to inhibit the human enzyme even at 100 μM. In the absence of a co-crystal structure of the human enzyme with 315, we superimposed the human structure (PDB: 1dq9)^34^ on Ref-315. Our structural analysis suggests that the imidazole side chain of His-752 in the human enzyme, which originates from a long wandering loop between Lα5 and Lα6 in the large domain, poses steric hindrance to the sulfonyl oxygen of 315. In the bacterial homologs, this bulky His-752 is substituted by Gly-264, which originates from a long alpha helix (analogous to Lα5) and does not clash with 315. The presence of Leu-851 in the human homolog in the place of Ala-363 in efHMGR also poses a steric clash with the benzoic acid moiety of 315 (Supplementary Fig. 5a). The prokaryotic Arg-257 which binds the benzoic acid group in 315 is replaced in the human enzyme by a lysine in the cis-peptide loop, eliminating one of two hydrogen bonds. The configuration of the nicotinamide ring for hydride transfer also changes, requiring other modifications to the contours of the active site pocket. These differences among others possibly resulted in the pronounced difference in inhibitor potency and selectivity of 315 towards the bacterial enzyme.

Previous research has shown that statins are weak inhibitors of the Class II HMG-CoA reductases, such as efHMGR, as compared to the nanomolar inhibition of Class I homologs. Kinetic analysis shows that 315 targeting the Class II homologs, especially efHMGR, is highly potent as compared to the statins. At 50 μM concentration, 315 achieves 80% inhibition of efHMGR as opposed to lovastatin achieving 32% inhibition. To understand the difference at the molecular level, the lovastatin bound pmHMGR structure (PDB: 1to2)^38^ was superimposed on the efHMGR structure and the interaction between lovastatin and efHMGR was analyzed. Since the active site residues are conserved between the two Class II homologs, the assumption is that lovastatin should interact with efHMGR in a similar fashion. Thus, instead of two hydrogen bond interactions between Arg-257 and the benzoic acid moiety in 315, only one H-bond is observed between the Arg-257 and the carboxylate group in lovastatin. As compared to four direct and four water-mediated hydrogen bond interactions between 315 and efHMGR, lovastatin makes three direct and two water mediated interactions with efHMGR (Supplementary Fig. 5b). The butylphenyl group of 315 is well accommodated in an extensive hydrophobic pocket formed by several residues from the flap domain as well as large domain of the enzyme. However, the decalin ring of lovastatin impedes the closure of flap domain and the extensive hydrophobic interaction is not observed.

Targeting pathogenic bacteria without disturbing the normal biome of the system is a positive for antibacterials to be used in therapeutic approaches. Common gut bacteria do not use the mevalonate pathway so inhibitors targeted to the HMGR of pathogenic Gram-positive bacterial species such as MRSA (methicillin-resistant *Staphylococcus aureus*) and (vancomycin-resistant *Enterococcus*) VRE would leave other beneficial bacteria undisturbed. Topical applications of these inhibitors may also prove useful in similar pathogenic infections. Our initial examination of 315 indicates that compounds based on this scaffold could well be highly selective to the bacterial HMGRs. Architectural differences in the active sites of the human and bacterial enzymes can be further exploited to optimize potency and selectivity of inhibitors based on the interactions we have observed between 315 and the two classes of HMG-CoA reductase. In addition, all the active site residues interacting with 315 are highly conserved in the gram-positive bacterial homologs (Supplementary Fig. 6), raising the prospect of specifically targeting gram-positive pathogens with a common inhibitor, thereby increasing their therapeutic reach.

## Methods

### Expression and purification of *E. faecalis* HMG-CoA reductase

The HMG-CoA reductase portion of the *E. faecalis mvaE* gene was cloned (residues 381-803) in pET28a vector and expressed with an N terminal 6X-Histidine tag in *Escherichia coli* BL21 (DE3) cells^17^. Cells were grown in the Luria Broth media in the presence of kanamycin (20 µg.ml^−1^) to 0.6–0.7 O.D_600_ at 37 °C and induced with 0.5 mM IPTG for 4 h. Cells were pelleted at 4000 g for 20 min and resuspended in lysis buffer A (10 mM imidazole, 20 mM HEPES pH 8.0, 300 mM NaCl) and subsequently lysed by a French pressure cell at 18,000 psi. The resulting cellular extract was centrifuged in an ultracentrifuge at 40,000 rpm for 1 hr. The supernatant (cytosol) was loaded onto a 2 ml column of nickel-NTA resin. The column was washed with buffer A and buffer B (50 mM imidazole, 20 mM HEPES pH 8.0, 300 mM NaCl), and eluted with buffer C (100 mM imidazole, 20 mM HEPES pH 8.0, 300 mM NaCl) and buffer D (500 mM imidazole, 20 mM HEPES pH 8.0, 300 mM NaCl) respectively. The fractions containing efHMGR, identified by denaturing polyacrylamide gel electrophoresis (10% SDS PAGE), demonstrated a single protein band at ~50 KDa. The fractions containing efHMGR were pooled together and precipitated by adding 3X volume of saturated ammonium sulfate (pH 7.0) with constant stirring at 4 °C for 1 h and subsequently pelleted down by centrifuging at 14,000 rpm for 20 min. The pellet was resuspended in 5 mM HEPES pH 8.0 and 300 mM NaCl. The protein concentration was measured with Bradford assay. The construct expresses 35 mg of N-terminal 6X-His tagged HMGR protein per liter of bacterial culture. Prior to crystallization, the protein was dialyzed against 5 mM HEPES pH 8.0, 300 mM NaCl in order to exchange out the ammonium sulfate.

### Crystallization

Initial crystallization conditions for the efHMGR apoenzyme (Ref-apo) were selected with the assistance of a robotic crystallization facility at the Hauptman-Woodward Medical Research Institute, Buffalo, New York. The initial hit was further optimized, yielding hexagonal P6_1_ crystals of efHMGR apoenzyme which were grown by sitting drop vapor diffusion method at 20 °C from solutions containing 3 µl of protein at 10 mg^.^ml^−1^ and 3 µl of 0.1 M MES pH 6.5, 50 mM calcium acetate, 16% PEG8000. The apoenzyme crystals grew to a size of 0.4 × 0.2 × 0.1 mm at 20 °C in 15–20 days. For data collection at 100 K, crystals were cryoprotected with a stepwise introduction of 20% PEG400 in crystallization buffer and flash frozen in liquid nitrogen.

For the ternary complex (Ref-ternary), efHMGR was co-crystallized with NADP^+^ and HMG-CoA using the sitting drop vapor diffusion technique at 20 °C. Plate-like C2 crystals appeared in the presence of 1.5 mM NADP^+^, 150 µM HMG-CoA, 0.1 M MES pH 6.7, 50 mM calcium acetate, and 8% PEG 8000. 10 mg.ml^−1^ of protein was used in these trials. Crystals appeared in 1–2 days and grew to a final size of 0.2 × 0.1 × 0.05 mm^3^. Crystals were cryoprotected with 35% glycerol in the crystallization buffer and flash frozen in liquid nitrogen.

The stock solution of the inhibitor, 315 was prepared by dissolving in 100% DMSO. The complex of efHMGR protein with inhibitor 315 (Ref-315) was co-crystallized in 25% PEG 4000, 0.17 M sodium acetate, 0.085 M Tris-HCl at pH 8.5 and 2% glycerol. The protein was at 14 mg.ml^−1^ in 5 mM HEPES pH 8.0, 300 mM potassium chloride, 650 μM 315 inhibitor, 1.6% DMSO, and trace amounts of ammonium sulfate and imidazole. Rod-like P2_1_2_1_2_1_ crystals appeared in few days and grew to a final size of 0.04 × 0.04 × 0.275 mm^3^. The crystals were cryoprotected in 15% PEG 400 in the crystallization buffer and flash frozen in liquid nitrogen.

### Data collection and processing

Native diffraction data to 2.25 Å for the Ref-apo crystals were collected at the SERCAT-ID beamline at Advanced Photon Source (APS, Argonne National Laboratory, Argonne, IL) using a CCD detector. The ref-apo crystals grew in the P6_1_ space group with four monomers in the asymmetric unit and cell dimensions of *a*, *b* = 168.7 Å, *c* = 120.2 Å, *α*, *β* = 90°, *γ* = 120°.

Single crystal diffraction data for the Ref-ternary crystals were collected at the SERCAT-BM beamline at the APS with a Rayonix (MAR 300) CCD detector. The data were indexed, integrated and scaled to 2.27 Å. The crystal lattice belongs to the monoclinic space group C2 with *a* = 241.3 Å, *b* = 62.6 Å, *c* = 171.9 Å, α = 90^°^, β = 133.5^°^, γ = 90° and four monomers per asymmetric unit.

The crystal structure of efHMGR complexed with inhibitor 315 was collected at the APS at the GM/CA beamline using a CCD detector. The data was indexed, integrated and scaled to 1.27 Å. The crystal lattice belongs to the orthorhombic space group P2_1_2_1_2_1_ with *a* = 72.5 Å, *b* = 81.1 Å, *c* = 152.9 Å, α, β, γ = 90^◦^ and two monomers per asymmetric unit. The data processing and reduction for Ref-apo, Ref-ternary and Ref-315 datasets were all done using HKL2000^39^ and the statistics are summarized in Table 1.

### Structure determination and refinement

Initial phase information for the efHMGR apoenzyme structure was obtained by molecular replacement using the *pm*HMGR dimer (PDB: 1qax, 38% sequence identity with efHMGR) as the initial search model for the two dimers in the asymmetric unit with Phaser^40^ in the CCP4 suite^41^. Residues 13 to 370 were modeled in the electron density map and a rigid body refinement was performed. This model was subsequently subjected to Resolve^42^, which involves a prime and switch density modification method to improve the electron density map. This initial model was first subjected to simulated annealing refinement in the CNS suite^43^. This model was subsequently corrected, expanded and refined against the 2.25 Å dataset by an iterative cycle of individual B-factor refinement, gradient minimization in REFMAC^44^ followed by model correction and solvent placement in Coot^45^. Partial occupancies, multiple conformations, calcium ions, 2-(N-morpholino) ethane sulfonic acid (MES) molecules and additional water molecules were added to the model. In the later stages, the model was subjected to a final refinement cycle in Phenix refine^46^. The final model consists of 4 monomers with residues 11-370 in chain A, 13-372 in chain B, 11-370 in chain C and 13-369 in chain D, 5 MES molecules, 2 sulfates, 4 ethylene glycol, 6 diethylene glycol, 10 calcium ions and 646 water molecules with R_work_ and R_free_ of 17.5 and 20.9 respectively. Post refinement the model was evaluated for errors using Procheck^47^ and Molprobity^48^.

The ligand bound efHMGR (Ref-ternary) structure was solved using the native apo-efHMGR as the search model. A rigid body refinement was carried out on the resulting model followed by Resolve^42^ to reduce model bias. The *F*_o_−*F*_c_ difference density calculated at this stage showed extra density in the active site of all of the monomers. This allowed us to model the substrate HMG-CoA, the product mevalonate and the cofactor NADP^+^. Additionally, large regions of extra density at the C-terminal end of the protein beyond residue 370 (the last visible residue in the apoenzyme) in two of the four monomers were observed and used to build the flexible flap comprising 51 amino acids for the C monomer (370– n421) and 34 residues for the B monomer (370 - 421). This initial model was first subjected to a few cycles of individual B factor refinement followed by simulated annealing refinement in CNS. The model was subsequently corrected and expanded by interactive model building and solvent placement in Coot, followed by few more cycles of restrained refinement in REFMAC and Phenix refine. The final model consists of 4 monomers with residues 13–370 in chain A, -4-421 in chain B, 0-421 in chain C and 13-369 in chain D modeled in the electron density map, 1 HMG-CoA, 1 mevalonate and 4 NADP^+^ molecules, 7 calcium ions, 9 acetate, 7 glycerol and 340 water molecules with a R_work_ and R_free_ of 19.6 and 23.9 respectively.

The efHMGR structure complexed with inhibitor 315 was solved using the unliganded efHMGR monomer as the search model. The structure was initially refined with Phenix using simulated annealing and later conjugate gradient methodologies. The unambiguous Fo-Fc density in the active site revealed the presence of the inhibitor in the active site. The model for the inhibitor was built in Chemdraw and energy minimized in the eLBOW module^49^ and then placed using the LigFit module of Phenix. Between refinements, the model structure was adjusted using COOT as indicated by the Fo-Fc difference densities. The final model consists of two monomers with residues 13-421 in chain A, 13-422 in chain B, 1 calcium ion, 2 inhibitor (315) molecules, 3 sulphate and 973 water molecules. Post refinement of all the models was evaluated for errors using Procheck^47^ and Molprobity^48^. All the models show good geometry with no residues in the outlier region of the Ramachandran plot. The data collection and refinement statistics is summarized in Table 1. The protein ligand interactions are analyzed using Ligplot^50^. The figures have been generated using Pymol^51^. The sequences have been aligned by the Tcoffee multiple sequence alignment program and visualized using Espript^52^.

### High-throughput screening of inhibitors at Southern Research Institute, Birmingham Alabama

Compound Dosing/Plating: Twenty five nanolitres (nL) of compounds in DMSO were dispensed into 384-welled clear non-binding surface treated plates, which resulted in final concentrations of 10 µM of compound in the assay.

Fifteen µL of HMG-CoA reductase reagent mix which included coenzyme A, NADP^+^, and mevalonate in the assay buffer was added to each well of the previously compound dosed 384-well plates. The reaction was initiated with the addition of 10 µL of HMG-CoA reductase diluted in assay buffer. The final concentrations in the reaction were 2 mM coenzyme A, 4 mM NADP^+^, 4 mM mevalonate, and 15 µg^.^ml^−1^ HMG-CoA reductase diluted in assay buffer (100 mM Tris-HCl (pH 8.0), 100 mM potassium chloride, 2% DMSO and 0.01% Tween 20). The test plate was immediately transferred to a Perkin Elmer Envision microplate reader and absorbance of NADPH was measured at 340 nm every 16 s for 160 s. Each plate had 64 control wells in the four outside columns with 32 containing the complete reaction mixture with carrier control (full reaction) and 32 in which the mevalonate had been left out^53^.

### IC_50_ measurement of Lovastatin and 315 against Class I (human) and Class II (*Enterococcus faecalis*) HMGR homologs

Inhibition of the oxidative acylation of mevalonate to HMG-CoA, by a common statin, Lovastatin, and 315, was investigated on HMG-CoA reductases from two different species using approximately similar reaction conditions. Briefly, reactions (100 μL) were set up in a 96-well, clear, flat bottom, and half-area plate (Corning Costar, Kennebunk, ME). First, 2.5 μL of the compound at a gradient concentration was added into wells followed by the master mixture. The master mixture composition is slightly different between the two classes of reductases. For efHMGR the master mixture composition is; 100 mM KCl, 100 mM Tris HCl, 1.5 mM Coenzyme A, 4.5 mM Mevalonate, 3 mM NADP^+^ and water to adjust the concentrations. For human HMG-CoA reductase the master mixture composition is; 50 mM NaCl, 100 mM Tris HCl, 1 mM EDTA, 5 mM DTT, 5 mM Coenzyme A, 6 mM Mevalonate, 5 mM NADP + and water to adjust the concentrations. Plate with the inhibitor and master mixture added was incubated at 37 ˚C for 3 min until the baseline is stable. Reaction was initiated by adding the enzyme with appropriate volumes to bring the final volume up to 100 μL. Reduction of NADP^+^ was monitored at 37˚C continuously by measuring the absorbance at 340 nm. Absorbance was read every 20 sec using a CLARIOstar Plus plate reader (BMG Labtech, Cary, NC). Early readings of the progress curves were used to calculate the initial slopes (IS). IC_50_ values were estimated through Michaelis-Menten fitting of the Inhibition % vs. inhibitor concentration data using non-linear regression in GraphPad Prism 9 (GraphPad Software, San Diego, CA, USA). Each experiment was carried out in triplicate under identical assay conditions. Assays in the absence of enzyme were used as experimental controls to determine the baseline slopes. Inhibition % was calculated according to the following equation.

1 $$Inhibition\, \% =\frac{I{S}_{HMGR+DMSO}-I{S}_{HMGR+Inhibitor}}{I{S}_{HMGR+DMSO}-I{S}_{EnzymeBuffer+DMSO}}x100 \%$$

### Measurement of the competitive inhibition (Ki) of the inhibitor, 315

The mevalonate oxidation assay was performed in a 200 μl reaction volume at 37 °C and the reaction was monitored by the appearance of NADPH at 340 nm. The standard assay conditions included 4 mM NADP^+^, 1 mM Coenzyme A, mevalonate at the following concentrations: 0.2 mM, 0.33 mM, 0.4 mM, 0.5 mM, 0.67 mM, 1 mM, 2 mM. The inhibitor (315) concentration was also varied as 5 μM, 7.5 μM and 10 μM. The assay buffer comprised of 100 mM KCl and 100 mM Tris-Cl pH 8.0. The mevalonate, NADP^+^ and inhibitor 315 were incubated in the assay buffer at 37 °C and the reaction was initiated by addition of the enzyme. The K_i_ value was determined with the mevalonate concentration varied as described above, keeping the other assay conditions standard. The double reciprocal plot of this inhibition study was obtained where 1/ initial rate was plotted as a function of 1/ substrate concentration and the data was fitted by linear regression in Graphpad Prism 9.0. The K_M_(apparent) and Km were obtained from this graph. The K_i_ value was calculated based on the equation:

$${{{{{{\rm{K}}}}}}}_{{{{{{\rm{i}}}}}}}={{{{{\rm{I}}}}}}/({{{{{{\rm{K}}}}}}}_{{{{{{\rm{M}}}}}}}({{{{{\rm{apparent}}}}}})/{K}_{M})\mbox{-}1.$$

#### Accession numbers

The accession number of the sequences used from the Uniprot database:

Q8DNS5: *Streptococcus pneumoniae* HMG-CoA Reductase

Q9FD86: *Staphylococcus aureus* HMG-CoA Reductase

Q9FD60: *Streptococcus pyogenes* HMG-CoA Reductase

A0A7ZD9J92: *Enterococcus hirae* HMG-CoA Reductase

Q9FD65: *Enterococcus faecium* HMG-CoA Reductase

Q8Y8R9: *Listeria monocytogenes* HMG-CoA Reductase

P13702: *Pseudomonas mevalonii* HMG-CoA Reductase

A9BQX8: *Delftia acidovorans* HMG-CoA Reductase

B4EKH5: *Burkholderia cenocepacia* HMG-CoA Reductase

Q9FD70: *Enterococcus faecalis* HMG-CoA Reductase

### Statistics and reproducibility

The kinetic data was analysed using GraphPad Prism Version 9.0 software. Data are presented as individual data points. Reproducibility was confirmed by performing three independent data points for Fig. 4b and two independent data points for Fig. 4c as described in the figure legend.

### Reporting summary

Further information on research design is available in the Nature Portfolio Reporting Summary linked to this article.

## Supplementary information

Supplementary Information

Description of Additional Supplementary Files

Supplementary Data 1

Supplementary Data 2

Reporting Summary

## Data Availability

All the reflection data and final coordinates for the crystallographic structures discussed have been submitted to the RCSB Protein Data Bank (http://pdb.org) under the following accession codes—*E. faecalis* HMG-CoA reductase apo (7M66), HMG-CoA, mevalonate and NADP^+^ bound (7M1Z) and compound 315 bound (7M3H), as described in Table 1. All source data behind the Kinetic graphs shown in the figures are presented in Supplementary Data 1 and Supplementary Data 2.

## References

[1] Hollenbeck BL, Rice LB (2012). Intrinsic and acquired resistance mechanisms in enterococcus. Virulence.

[2] Paulsen IT (2003). Role of mobile DNA in the evolution of vancomycin-resistant Enterococcus faecalis. Science.

[3] Simor AE (2013). Staphylococcus aureus and vancomycin-resistant Enterococcus and of Clostridium difficile infection in Canadian hospitals. Infect. Hosp. Epidemiol..

[4] Goldstein JL, Brown MS (1990). Regulation of the mevalonate pathway. Nature.

[5] Johnson EA, Schroeder WA (1996). Microbial carotenoids. Adv. Biochem. Eng. Biotechnol..

[6] Reusch VM, Salton MRJ (1984). Lipopolymers, isoprenoids, and the assembly of the gram-positive cell wall. Crit. Rev. Microbiol..

[7] Pelz A (2005). Structure and biosynthesis of staphyloxanthin from Staphylococcus aureus. J. Biol. Chem..

[8] Wilding EI (2000). Essentiality, expression, and characterization of the class II 3-hydroxy-3-methylglutaryl coenzyme a reductase of Staphylococcus aureus. J. Bacteriol..

[9] Wilding EI (2000). Identification, evolution, and essentiality of the mevalonate pathway for isopentenyl diphosphate biosynthesis in gram-positive cocci. J. Bacteriol..

[10] Lichtenthaler HK (2000). Non-mevalonate isoprenoid biosynthesis: Enzymes, genes and inhibitors.. Biochem. Soc. Trans..

[11] Wagner WP, Helmig D, Fall R (2000). Isoprene biosynthesis in Bacillus subtilis via the methylerythritol phosphate pathway. J. Nat. Prod..

[12] Ducarmon, Q. R. et al. Gut microbiota and colonization resistance against bacterial enteric infection. *Microbiol. Mol. Biol. Rev*. **83**, 716299 (2019). 10.1128/MMBR.00007-19 PMC6710460 31167904

[13] Bochar, D. A., Freisen, J., Stauffacher, C. V. & Rodwell, V. W. *Comprehensive Natural Products Chemistry* (Elsevier, 1999).

[14] Hedl M, Tabernero L, Stauffacher CV, Rodwell VW (2004). Class II 3-hydroxy-3-methylglutaryl coenzyme areductases?. J. Bacteriol..

[15] Istvan ES (2001). Bacterial and mammalian HMG-CoA reductases: related enzymes with distinct architectures. Curr. Opin. Struct. Biol..

[16] Hedl M (2002). Enterococcus faecalis acetoacetyl-coenzyme A thiolase/3-hydroxy-3-methylglutaryl-coenzyme A reductase, a dual-function protein of isopentenyl diphosphate biosynthesis. J. Bacteriol..

[17] Hedl, M. *Isopentenyl Pyrophosphate Biosynthesis in Bacteria: Genes and Enzymes of the Mevalonate Pathway* (Purdue University, 2003).

[18] Miller BR, Kung Y (2018). Structural features and domain movements controlling substrate binding and cofactor specificity in Class II HMG-CoA reductase. Biochemistry.

[19] Peacock RB (2019). Structural and functional characterization of dynamic oligomerization in burkholderia cenocepacia HMG-CoA reductase. Biochemistry.

[20] Steussy CN (2013). A novel role for coenzyme a during hydride transfer in 3-hydroxy-3- methylglutaryl-coenzyme a reductase. Biochemistry.

[21] Lawrence CM, Rodwell VW, Stauffacher CV (1995). Crystal structure of Pseudomonas mevalonii HMG-CoA reductase at 3.0 angstrom resolution. Science.

[22] Liu Y, Eisenberg D (2002). 3D domain swapping: as domains continue to swap. Protein Sci..

[23] Rousseau F, Schymkowitz JWH, Itzhaki LS (2003). The unfolding story of three-dimensional domain swapping. Structure.

[24] Istvan EvaS, Palnitkar Maya, Buchanan, Susan K, Deisenhofer J (2000). Crystal structure of the catalytic portion of human HMG-CoA reductase: insights into regulation of activity and catalysis. EMBO J..

[25] Vögeli B, Shima S, Erb TJ, Wagner T (2019). Crystal structure of archaeal HMG-CoA reductase: insights into structural changes of the C-terminal helix of the class-I enzyme. FEBS Lett..

[26] Ragwan ER, Arai E, Kung Y (2018). New crystallographic snapshots of large domain movements in bacterial 3-Hydroxy-3-methylglutaryl coenzyme A reductase. Biochemistry.

[27] Huang Y, Gao M, Su Z (2018). Exploring the roles of proline in three-dimensional domain swapping from structure analysis and molecular dynamics simulations. Protein J..

[28] Gil G, Faust JR, Chin DJ, Goldstein JL, Brown MS (1985). Membrane-bound domain of HMG CoA reductase is required for sterol-enhanced degradation of the enzyme. Cell.

[29] Haines BE, Steussy CN, Stauffacher CV, Wiest O (2012). Molecular modeling of the reaction pathway and hydride transfer reactions of HMG-CoA reductase. Biochemistry.

[30] Baker PJ, Britton KL, Rice DW, Rob A, Stillman TJ (1992). Structural consequences of sequence patterns in the fingerprint region of the nucleotide binding fold. Implications for nucleotide specificity. J. Mol. Biol..

[31] Kim D-Y, Stauffacher CV, Rodwell VW (2000). Dual coenzyme specificity of Archaeoglobus fulgidus HMG-CoA reductase. Protein Sci..

[32] Tabernero L, Bochar DA, Rodwell VW, Stauffacher CV (1999). Substrate-induced closure of the flap domain in the ternary complex structures provides insights into the mechanism of catalysis by 3-hydroxy-3-methylglutaryl-CoA reductase. Proc. Natl Acad. Sci. USA.

[33] Friesen JA, Lawrence M, C., Stauffacher CV, Rodwell VW (1996). Structural determinants of nucleotide coenzyme specificity in the distinctive dinucleotide binding fold of HMG-CoA reductase from Pseudomonas mevalonii. Biochemistry.

[34] Istvan ES, Deisenhofer J (2000). The structure of the catalytic portion of human HMG-CoA reductase. Biochim. Biophys. Acta.

[35] Qureshi N, Dugan RE, Cleland WW, John W, Porter (1976). Kinetic analysis of the individual reductive steps catalyzed by β-Hydroxy-β-methylglutaryl-coenzyme A reductase obtained from yeast. Biochemistry.

[36] Veloso D, Cleland WW, Porter JW (1981). pH properties and chemical mechanism of action of 3-Hydroxy-3-methylglutary 1 coenzyme A reductase. Biochemistry.

[37] Li D (2012). Structure-based design and screen of novel inhibitors for class II 3-hydroxy-3-methylglutaryl coenzyme a reductase from Streptococcus pneumoniae. J. Chem. Inf. Model..

[38] Tabernero L, Rodwell VW, Stauffacher CV (2003). Crystal structure of a statin bound to a class II hydroxymethylglutaryl-CoA reductase. J. Biol. Chem..

[39] Otwinowski Z, Minor W (1997). Processing of X-ray diffraction data collected in oscillation mode. Methods Enzymol..

[40] McCoy AJ (2007). Phaser crystallographic software. J. Appl. Crystallogr..

[41] CCP4. (1994). Collabortive computational project number 4. The CCP4 suite of programs for protein crystallography. Acta Crystallogr. Sect. D. Biol. Crystallogr..

[42] Terwilliger T (2004). SOLVE and RESOLVE: automated structure solution, density modification, and model building. J. Synchrotron Radiat..

[43] Pannu NS (1998). Crystallography & {NMR} system: a new software suite for macromolecular structure determination. Acta Crystallogr. D. Biol. Crystallogr..

[44] Murshudov GN, Vagin AA, Dodson EJ (1997). Refinement of macromolecular structures by the maximum-likelihood method. Acta Crystallogr. Sect. D: Biol. Crystallogr..

[45] Emsley P, Cowtan K (2004). Coot: Model-building tools for molecular graphics. Acta Crystallogr. Sect. D. Biol. Crystallogr..

[46] Adams PD (2010). PHENIX: A comprehensive Python-based system for macromolecular structure solution. Acta Crystallogr. Sect. D. Biol. Crystallogr..

[47] Laskowski, R. A., MacArthur, M. W. & Thornton, J. M. PROCHECK: validation of protein-structure coordinates. *Int. Tables Crystallogr*.10.1107/97809553602060000882 (2012).

[48] Chen VB (2010). MolProbity: All-atom structure validation for macromolecular crystallography. Acta Crystallogr. Sect. D. Biol. Crystallogr..

[49] Moriarty NW, Grosse-Kunstleve RW, Adams PD (2009). Electronic ligand builder and optimization workbench (eLBOW): A tool for ligand coordinate and restraint generation. Acta Crystallogr. Sect. D. Biol. Crystallogr..

[50] Laskowski RA, Swindells MB (2011). LigPlot+: Multiple ligand-protein interaction diagrams for drug discovery. J. Chem. Inf. Model..

[51] DeLano WL (2002). Pymol: An open-source molecular graphics tool. CCP4 Newsl. Protein Crystallogr.

[52] Gouet P, Courcelle E, Stuart DI, Métoz F (1999). ESPript: Analysis of multiple sequence alignments in PostScript. Bioinformatics.

[53] Stauffacher, C. V. *High Throughput Screen to Identify Compounds that Inhibit Class II HMG-CoA Reductases—Primary Screen*. https://pubchem.ncbi.nlm.nih.gov/bioassay/1066 (2008).

