# Supplementary material for: Targeting Enterococcus faecalis HMG-CoA reductase with a non-statin inhibitor
Source: Commun Biol. 2023 Apr 3;6:360. doi: 10.1038/s42003-023-04639-y (PMC10070635; doi:10.1038/s42003-023-04639-y)
Supplement: Supplementary file 1 — Supplementary Information [file 42003_2023_4639_MOESM1_ESM.pdf]

Supplementary Information

Supplementary Figure 1

Tetramer architecture of Apo and liganded efHMGR

a. The Apo efHMGR (Ref-apo**)** tetramer: The four monomers that arranged as dimer of dimers are colored green (A), blue (B), magenta (C) and yellow (D) respectively. The dimer-dimer interface interactions involve a calcium ion (orange) present in the crystallization buffer. The inset figure shows the dimer-dimer interaction at the AC interface. The residues Ala-341, Thr-343 and Glu-347 from both the monomers A and C coordinate with the calcium ion present in the centre. Water molecules are shown as red spheres and the metal coordination interactions and hydrogen bond interactions as red dashes and distances in Å.

b. Tetramer architecture in the efHMGR liganded complex (Ref-ternary) . In this case, the dimer-dimer interactions are seen primarily between monomer B and monomer C involving the flap domain (red). The inset figure shows that Arg-404 of monomer C (colored magenta) hydrogen bonds with Asp-338 of monomer B (colored blue) and Gln-31 of monomer A (colored green). The hydrogen bond interactions are shown as red dashes and the distances are in Å.

a

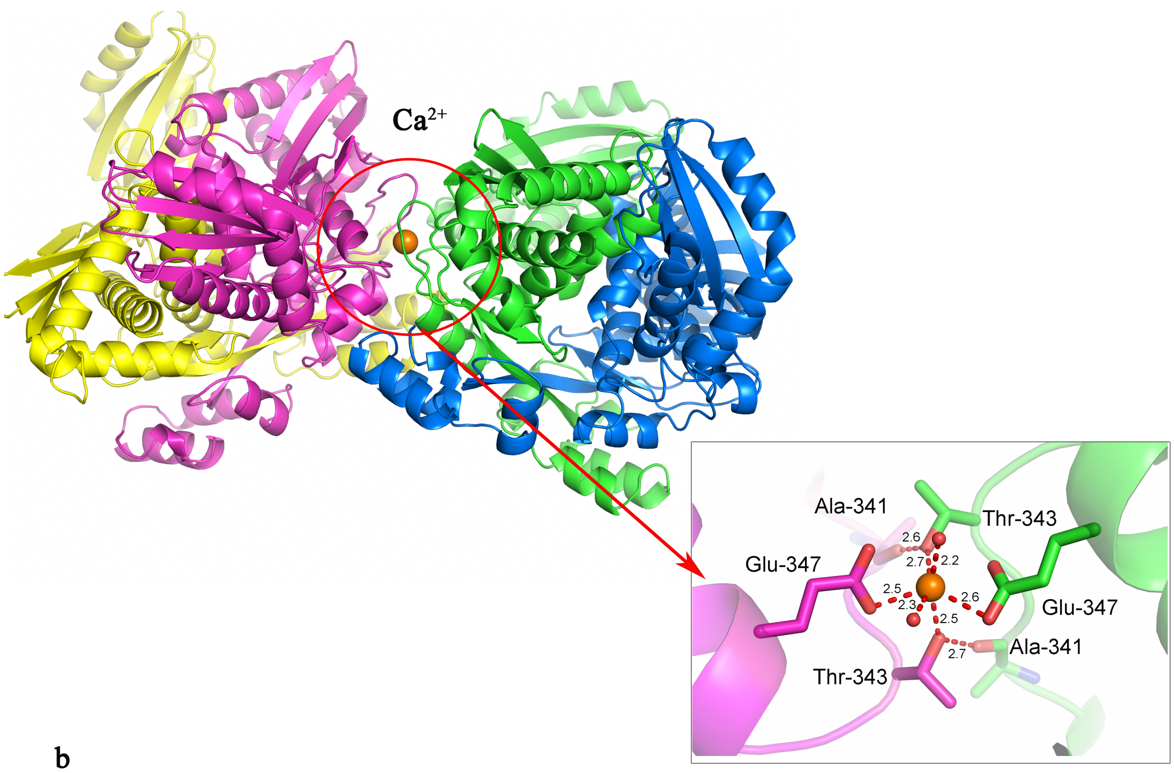

Ca^{2+}
Ala-341
Thr-343
2.6
2.7
2.2
Glu-347
2.5
2.3
2.6
2.5
Glu-347
2.7
Ala-341
Thr-343

b

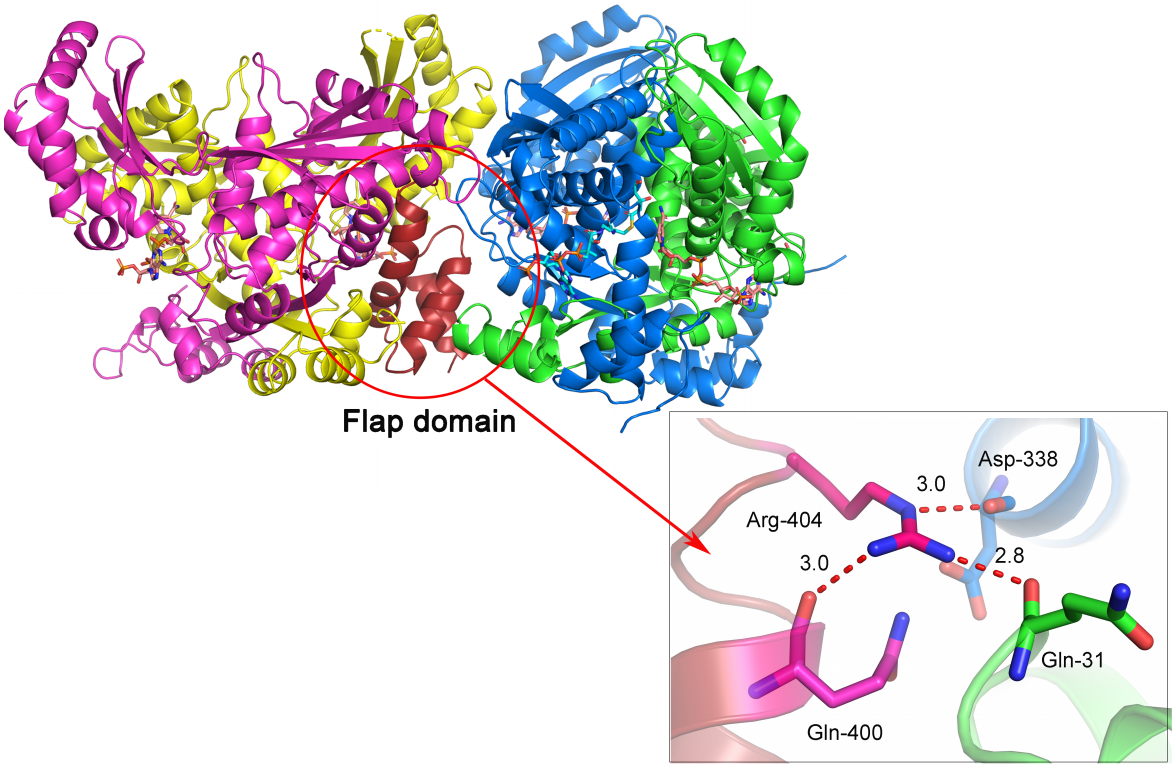

Flap domain
Asp-338
3.0
Arg-404
3.0
2.8
Gln-31
Gln-400

Supplementary Figure 2

Domain swapping in efHMGR, pmHMGR and human HMGR

In the Class I human HMGR (green) and Class II efHMGR (magenta), the N- terminal domain of one monomer makes a cross over and interact with the second monomer. However, in Class II pmHMGR (blue), the N terminal domain double backs on itself and interact with the same monomer. In all cases, the second monomer is colored gray.

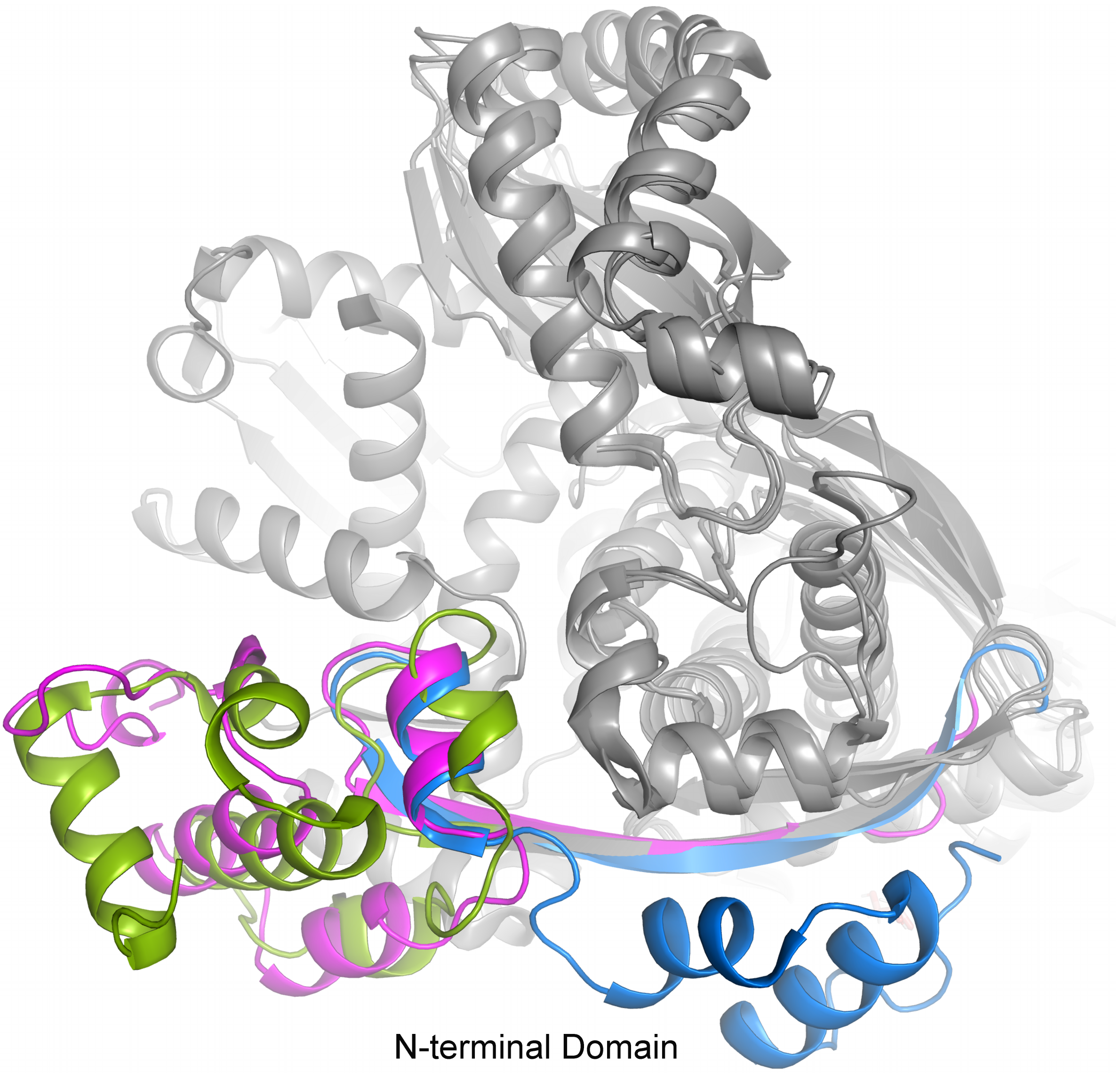

Supplementary Figure 3

Ligplot diagram elucidating the interactions between efHMGR and HMG-CoA, Mevalonate and $NADP^+$ respectively

a. HMG-CoA: HMG-CoA binds in an extended conformation primarily in the large domain of the enzyme. The adenosine diphosphate (ADP) moiety of CoA interacts with the positively charged pocket near the enzyme surface while the HMG moiety is located deep in the active site at the other end. A $\pi - \pi$ stacking interaction is observed between Tyr-15 and the adenine moiety of CoA. The adenosine-ribose -phosphate group is stabilized by direct hydrogen bond interactions with His-70 (2.8Å) and Lys-98 (2.9Å) along with several water-mediated interactions. The diphosphate moiety of ADP is stabilized by interactions with Lys-233 (3.2Å) and a glycerol molecule (3.1Å). The pantothenic part of CoA extends between the two along the active site with several stabilizing interactions observed between the enzyme and HMG-CoA. The nitrogen N4P, of the 1st isopeptide bond is at a hydrogen bond distance to hydroxyl group of Ser88 (2.8Å). The CoA thioester sulphur atom lies close to the catalytic His-376 from the first helix of the flap domain. A hydrogen bond interaction is seen between Asn-95 side chain amide group, and the oxygen atom (O9P) of the pantothenate group (3.1Å). A water mediated hydrogen bond interaction is also observed between the side chain amide group of Gln-359 and the oxygen (O2) of pantothenic group. The HMG/mevalonate binding pocket lies at the dimer interface in a relatively protected cleft and is conserved across all HMGRs. Two hydrogen bond interactions (2.5 Å and 2.9Å) are observed between the guanidino group of Arg-257 and the carboxylate group of HMG. The thioester bond is intact and the carbonyl oxygen of the thioester is rotated 180° as compared to the analogous pmHMGR structure (PDB: 1qax) in the presence of HMG-CoA and NAD^+. In this configuration, the thioester oxygen lies is ~5Å away from the catalytic glutamate residue (Glu-86) and lysine residue (Lys-263) . Several water mediated interactions along with the hydrogen bond and hydrophobic interactions stabilize HMG-CoA in the large domain of the enzyme.

b. $NADP^+$: The adenine ring of $NADP^+$ lies near the surface of the enzyme, stabilized by stacking interaction with Arg152 (3.5Å) and a hydrophobic interaction with Val323 (3.5Å). The N1A atom and the 6-amino moeity of adenine interact with the main chain amide (3.3Å) and the side chain carboxyl group of Asp-179 (3.2 Å) respectively via hydrogen bonds. The pyrophosphate moiety hydrogen bonds with the main chain amide of Met-181(3.2Å) and

Gly-182 (3.1Å) residues located in the small domain. The 2'-OH of the nicotinamide ribose group is stabilized by hydrogen bond interactions with side chain carboxyl oxygen of Asp-279 (2.8 Å). The nicotinamide-ribose-oxygen hydrogen bonds with the side chain amide group of Asn-184 (3.1Å). The carboxamide group of the nicotinamide ring is stabilized by hydrogen bond interactions with the side chain carboxamide moeity of Asn-213 (3.1Å and 2.8Å respectively). The interactions that stabilize the adenine-ribose-phosphate group of $NADP^+$ are discussed in the main text. In all the figures, cyan sphere represents water molecules, green dashes represent hydrogen bond interactions (between 2.3 to 3.3Å distance) and brick red spoked arcs represent hydrophobic interactions. Ligand bond is shown in purple, and atoms are shown as black for carbon, red for oxygen, yellow for sulpher and blue for nitrogen.

c. Mevalonate: The carboxylate group of mevalonate maintains the direct hydrogen bond interactions (2.7Å and 3.0Å respectively) with the guanidinium group of Arg-257. Several hydrophobic residues such as Ala-363, Ile-372, Leu-379 surround the methyl group of the mevalonate. The terminal hydroxyl group of mevalonate interacts with the catalytic lysine (Lys-263, 3.1Å) and glutamate (Glu-86, 2.6Å) residues of the enzyme.

d. The mevalonate represented by magenta sticks is encased in 2Fo-Fc density at 1.0 σ.

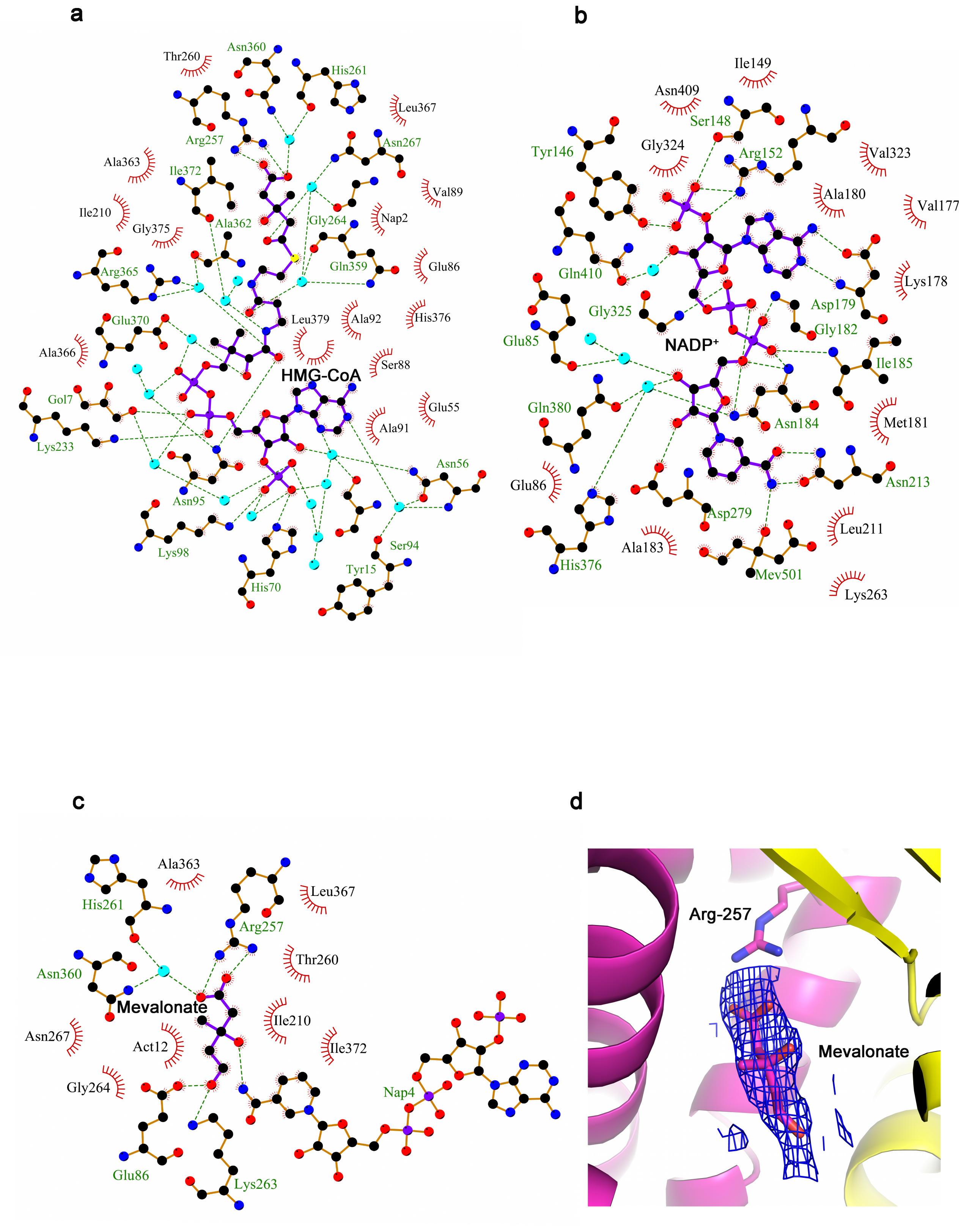
a
HMG-CoA
Thr260
Asn360
His261
Leu367
Arg257
Asn267
Ala363
Ile372
Val89
Ile210
Gly375
Ala362
Gly264
Nap2
Arg365
Gln359
Glu86
Glu370
Leu379
Ala92
His376
Ala366
Ser88
Gol7
Glu55
Lys233
Ala91
Asn56
Asn95
Lys98
Ser94
Tyr15
His70
b
NADP+
Ile149
Asn409
Ser148
Tyr146
Gly324
Arg152
Val323
Ala180
Val177
Gln410
Lys178
Gly325
Asp179
Glu85
Gly182
Ile185
Gln380
Asn184
Met181
Glu86
Asn213
Asp279
Leu211
Ala183
His376
Mev501
Lys263
c
Mevalonate
Ala363
His261
Leu367
Arg257
Thr260
Asn360
Asn267
Ile210
Act12
Ile372
Gly264
Nap4
Glu86
Lys263
d
Arg-257
Mevalonate

Supplementary Figure 4

Residues in the hinge region undergo conformational changes upon flap domain closure in efHMGR. Salt bridge interactions between Arg-365 and Glu-370 at the hinge region (residues 368-370) are observed during flap domain closure in both the liganded and 315 bound forms of efHMGR. In the presence of HMG-CoA, Glu-370 further interacts with the ligand and subsequent residues of the flap domain, Gly-371 and Gly-375 via water-mediated hydrogen bond interactions. A water mediated hydrogen bond interaction is also observed between the arginine side chain and the carbonyl oxygen of the pantothenic group in HMG-CoA. The water molecules are represented as red spheres and hydrogen bond interactions are shown as red dashes and the distances are in Å.

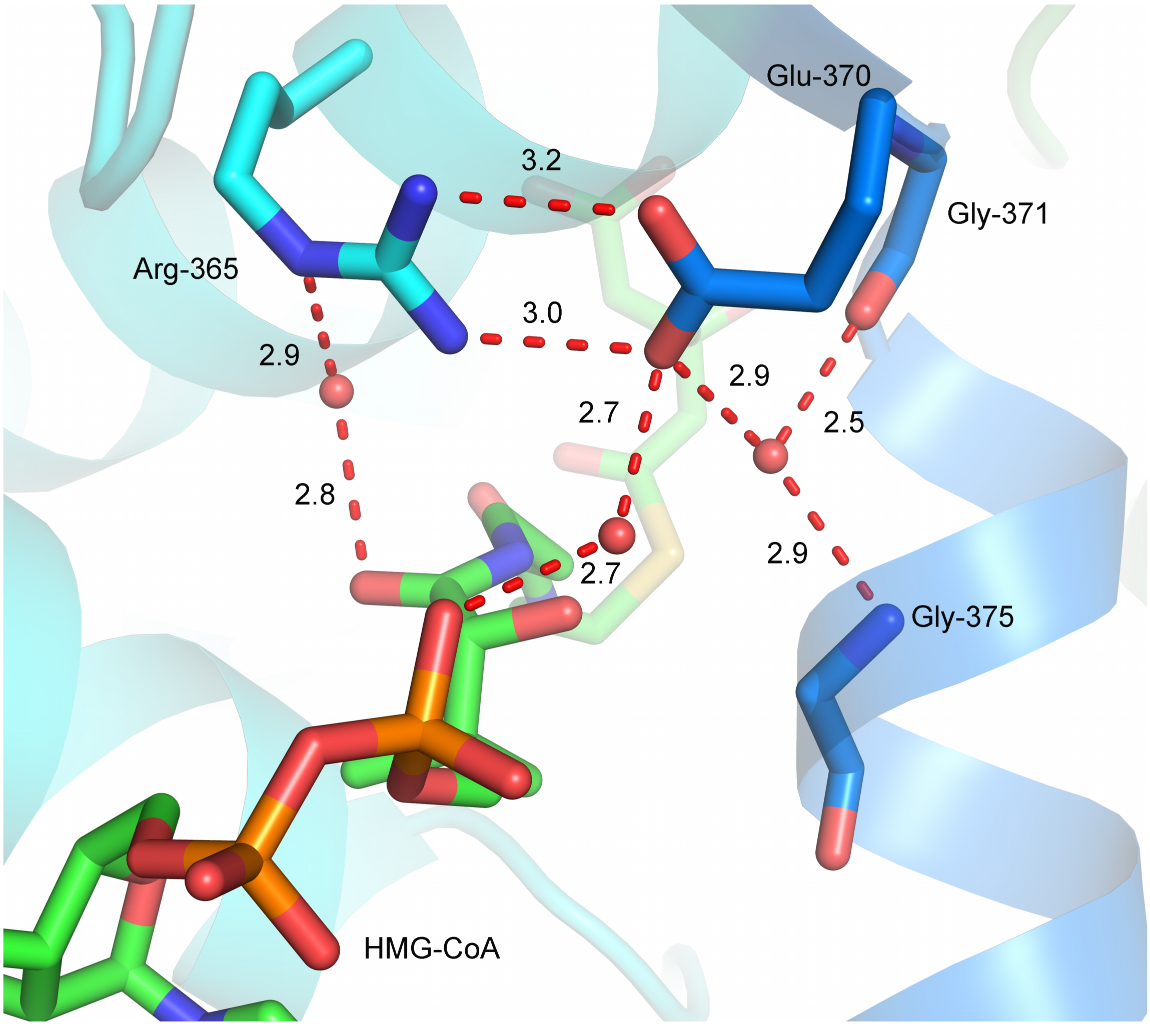

Supplementary Figure 5

a. Interactions of 315 with the human enzyme. The human HMGR (PDB: 1dq8) was superimposed on the Ref-315 structure and the plausible interactions between 315 and the human enzyme was analyzed. His-752 in the human enzyme poses a steric clash to the sulphonyl oxygen of 315. Similarly, Leu-851 also poses steric hindrance to the benzoic acid moiety of 315. These critical interactions, among other changes in the active site environment, could lower the effectiveness of 315 as an inhibitor of the Class I human enzyme.

b. Interactions of Lovastatin with efHMGR. Lovastatin bound pmHMGR structure (PDB:1to2) was superimposed on the efHMGR (Ref-apo) structure. Lovastatin makes three direct and two water-mediated hydrogen bond interactions with various active site residues (Asn-213, Arg-257, Lys-263 and Asn-267) of efHMGR. The potential hydrogen bond interactions are shown as red dashes and the water molecules as red spheres.

a

b

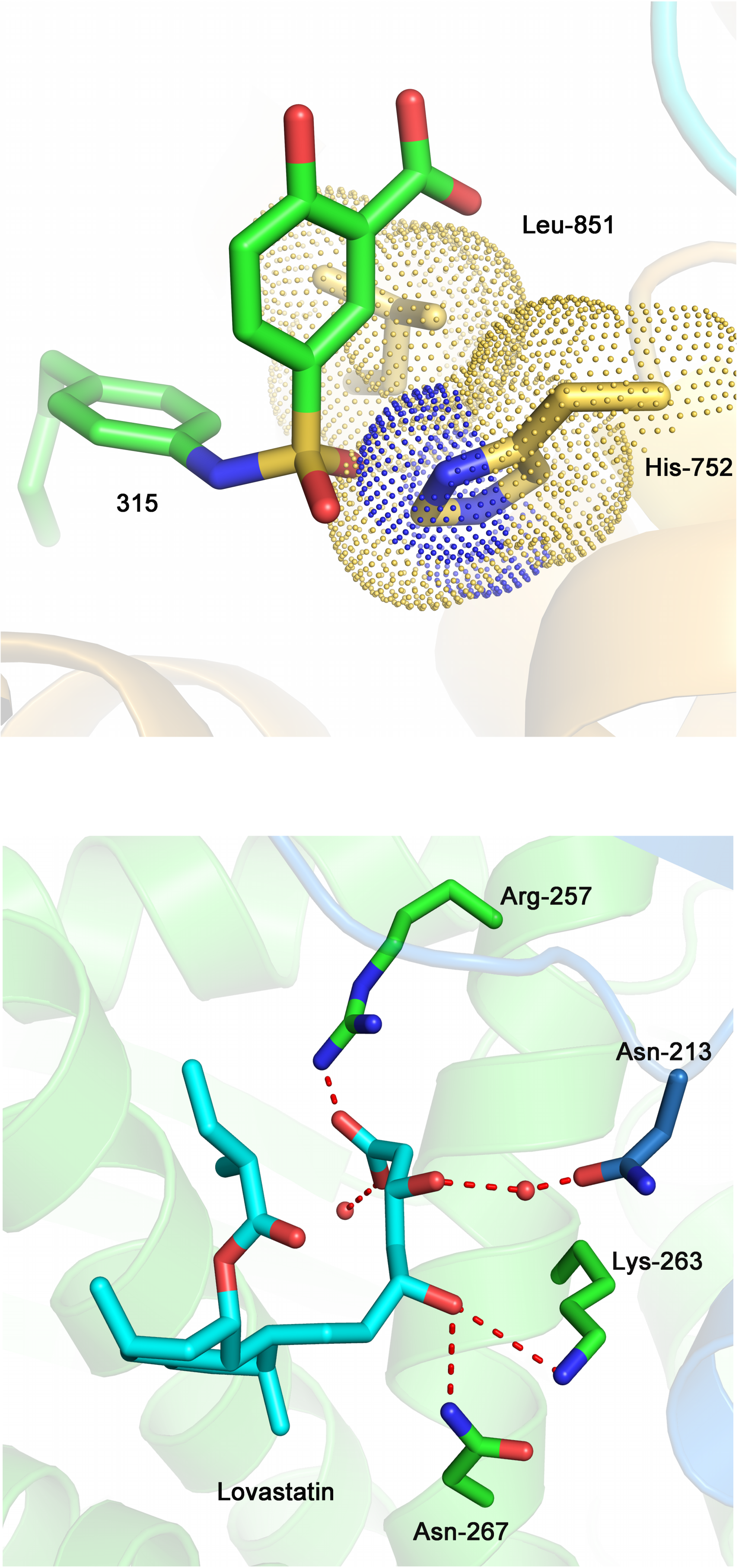

Supplementary Figure 6

Residues that interact with 315 are conserved in the pathogenic bacterial HMGR homologs. Amino acid sequence alignment of representative gram-positive pathogenic bacterial HMGRs including that *of Staphylococcus aureus, Listeria monocytogenes, Streptococcus pneumoniae, Streptococcus pyogenes* and *Enterococcus faecalis* is shown. *Burkholderia cenocepacia,* which is a gram-negative pathogenic bacterium is also included. This alignment suggests that 315 binding residues (marked with blue asterix) are conserved across the pathogenic HMGRs. The semiconserved residue are marked with green asterix.

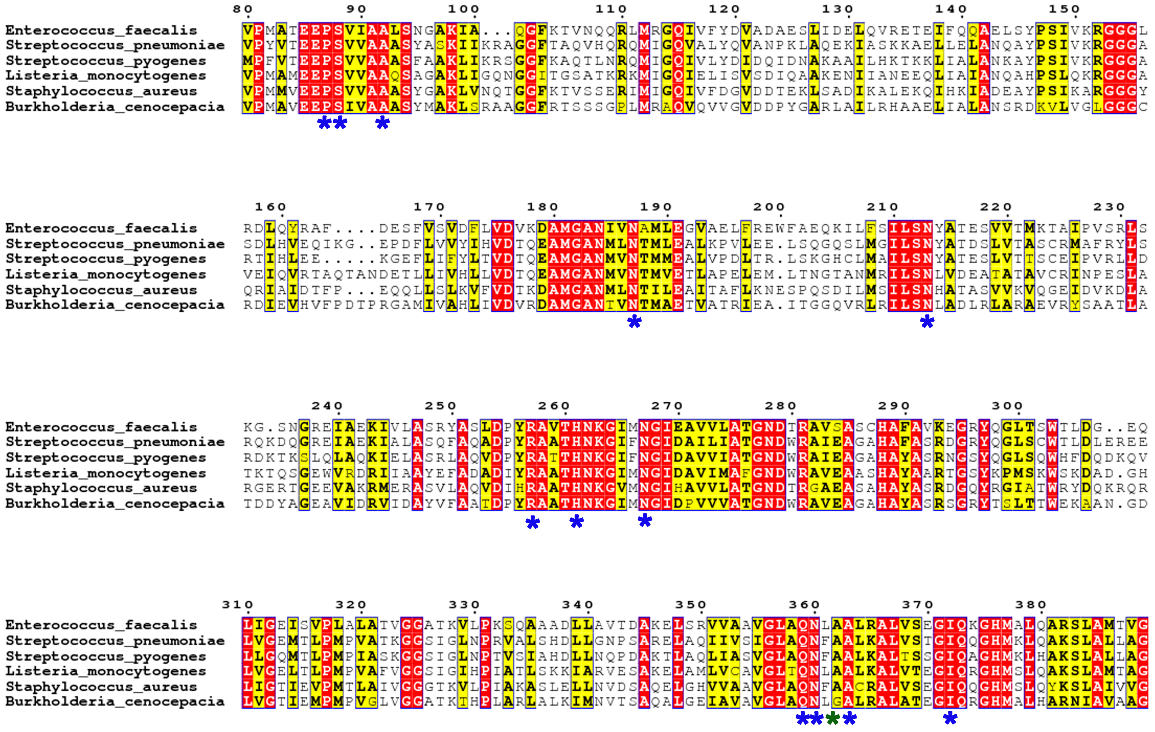

Supplementary Note 1

Interactions at the dimer-dimer interface of the apoenzyme

The tetramer in the asymmetric unit of the apoenzyme is comprised of two dimers (AB and CD). The dimer-dimer interface is observed primarily between the monomer A and C with a buried surface area of 390 Å^2 and is mediated by a calcium ion from the crystallization buffer (Supplementary Figure 1a). However, additional interactions are observed between monomers B and C, B and D, and A and D as well. The calcium ion present at the AC interface coordinates with the side-chain carboxyl oxygen of Glu-347 and side-chain hydroxyl group of Thr-343 from monomer A and the same set of residues from monomer C as well. Additional hydrogen bond interactions between residues Thr-343 (A) and Ala-341 (C), Glu-75 (A) and Lys 346 (C) are also observed (Supplementary Figure 1a inset). Salt bridge interactions at the interface are also observed between Lys-178 (A) and Asp-77 (C). The presence of a two-fold symmetry axis at the AC interface makes this interface symmetric wherein equivalent amino acids from both the subunits are engaged in similar interactions. Thus, same set of interactions are observed between Thr-343 (C) and Ala-341 (A), Glu-75 (C) and Lys-346 (A), and Lys-178 (C) and Asp-77 (A) respectively.

Interactions at the dimer-dimer interface of the liganded form

In the liganded structure, the dimer-dimer interface is different when compared to the apoenzyme. Here, the major interface is observed between the flap domain of monomer B and large domain of monomer C with an interface area of 635 Å^2 (Supplementary Figure 1b). The guanidinium group of Arg-404 of monomer C hydrogen bonds with carbonyl oxygen of Asp-338 (3.0 Å) from monomer B and carbonyl oxygen of Gln-31 (2.8Å) of monomer A (Supplementary Figure 1b, inset). The side-chain carboxyl group of Glu-75 (B) hydrogen bonds with main chain amide group of Lys-233 (2.52Å) and side-chain hydroxyl group of Ser-233 (2.55Å). Smaller interfaces are also formed between monomers B and D (295.6 Å^2), A and C (125 Å^2), and A and D (21.5 Å^2) respectively.

Supplementary Note 2

Domain Swapping in efHMGR

Our data suggests that the "dimerization element" between αC and βA in efHMGR has a significant role in the domain swap. Uniquely, the aforementioned element contains a sequence EN$\underline{\mathbf{Q}}_{57}I\underline{\mathbf{S}}_{59}$$X_3$L/P with substitutions glutamine and serine (underlined) for valine and glycine, otherwise present in most other bacterial homologues (Figure 1e). All the three efHMGR structures reported here suggest that Gln-57, Ser-59 (in the ENQIS loop) and Glu-60 make significant interactions, which stabilizes this crossover. H-bond interactions are observed between Gln-57 side chain amide group and Ser59 main chain carbonyl oxygen atom, which positions the Ser-59 side chain hydroxyl group at a H-bonding distance to the carbonyl oxygen of Phe-40 (in the αB helix) of the same monomer (Figure 1c). Moreover, the Glu-60 side chain carboxyl group makes H-bond interactions with Ala-44 and Leu-45 backbone amide groups of the same monomer (2.8Å and 3.0Å). The side chain carboxyl oxygen atom of Glu-62 (2.8Å), Glu-39 (2.8Å) and main chain oxygen atom of Asn-42 (2.7Å) of the same monomer participate in several H-bond interactions with the ε-amino group of Lys-332 from the C-terminal domain of the adjacent monomer (Figure 1c). These interactions near the crossover point at the N terminus stabilize the domain swapping in the efHMGR dimer.

In other prokaryotic HMGR enzymes including pmHMGR (PDB: 1qax)[32], *Streptococcus pneumoniae* HMGR (PDB: 5wpj)[18], *Delftia acidovorans* HMGR (PDB: 6eeu)[26] and *Burkholderia cenocepacia* HMGR (PDB: 6p7k)[19], the ENQIS sequence is substituted by the ENVXG sequence which makes no such interactions. In pmHMGR, DaHMGR and BcHMGR, which swap only 16 residues , a proline residue (Pro-43/Pro-43/Pro-64) is present at the hinge region that precedes the ENVXG sequence. This proline residue introduces conformational constraints which force the rest of the N-terminal domain to fold back on itself and thereby prevents an entire domain swap. Hydrophobic interactions between Met-44/Leu-44/Leu-65 , Ala-47/Ala-47/Ala-68 and Val-54/Val-54/Val-65 further stabilize the partially swapped conformation in the other bacterial homologs (Figure 1d).

Supplementary Note 3

Flap domain orientations upon ligand binding in bacterial homologs

Briefly, the consensus is that the binding of both NAD/P^+ and HMG-CoA or its derivatives trigger full closure of the flap domain over the active site, as seen in the pmHMGR and human enzymes [20,24,32]. However, there are conflicting reports regarding the orientation of the flap domain when either HMG-CoA or NAD/P^+ alone is bound. The cofactor bound structures of several prokaryotic (pmHMGR)[18,20] and eukaryotic HMGR homologs [24] suggest that NAD/P^+ binding alone does not trigger the flap domain ordering and closure. The only cases where flap was seen upon NAD/P^+ binding was in *Streptococcus pneumoniae* and *Delftia acidovorans* HMGR. In SpHMGR, the flap was ordered but displayed an open conformation. In the DaHMGR structure,[26] the flap domain was ordered but "flipped upside down".

The flap domain was disordered in pmHMGR with only HMG-CoA in the active site (PDB: 4i6a) [20]. In 2018, Miller *et.al*[18] reported the structure of *Streptococcus pneumoniae* HMGR (SpHMGR, PDB: 5wpk), where they showed that in the presence of HMG-CoA, the flap domain occupied a partially closed conformation, with the catalytic His-378 placed far away from the active site Ser-83. However, the most recent structure of coenzyme-A bound form of *Burkholderia cenocepacia* HMGR (PDB: 6p7k)[19] also shows a disordered flap domain.
