# Supplementary material for: Targeting Enterococcus faecalis HMG-CoA reductase with a non-statin inhibitor
Source: Commun Biol. 2023 Apr 3;6:360. doi: 10.1038/s42003-023-04639-y (PMC10070635; doi:10.1038/s42003-023-04639-y)
Supplement: Supplementary file 2 — Description of Additional Supplementary Files [file 42003_2023_4639_MOESM2_ESM.pdf]

Description of Additional Supplementary Files

File name: Supplementary Data 1
Description: Microsoft Excel file containing the raw data used for Figure 4b.

File name: Supplementary Data 2
Description: Microsoft Excel file containing the raw data used for Figure 4c.
